# A Self‐Sustaining Antioxidant Strategy for Effective Treatment of Myocardial Infarction

**DOI:** 10.1002/advs.202204999

**Published:** 2022-12-25

**Authors:** Quan Sun, Hongqin Ma, Jiaxiong Zhang, Baiyang You, Xiaohui Gong, Xiaolin Zhou, Jin Chen, Guogang Zhang, Jia Huang, Qiong Huang, Yurong Yang, Kelong Ai, Yongping Bai

**Affiliations:** ^1^ Department of Geriatric Medicine Coronary Circulation Center Xiangya Hospital Central South University Changsha Hunan P.R. China; ^2^ National Clinical Research Center for Geriatric Disorders Xiangya Hospital Central South University Changsha Hunan P.R. China; ^3^ Xiangya School of Pharmaceutical Sciences Central South University Changsha Hunan P.R. China; ^4^ Hunan Provincial Key Laboratory of Cardiovascular Research Xiangya School of Pharmaceutical Sciences Central South University Changsha Hunan P.R. China; ^5^ Cardiac Rehabilitation Center Department of Rehabilitation Xiangya Hospital of Central South University Changsha Hunan P.R. China; ^6^ Department of Cardiology The Third Xiangya Hospital Central South University Changsha Hunan P.R. China; ^7^ Department of Pharmacy Xiangya Hospital Central South University Changsha 410008 China

**Keywords:** angiogenesis, anti‐inflammatory, fibrosis, heart failure, myocardial infarction, passively targeted mitochondria, ROS‐scavenging

## Abstract

Myocardial infarction (MI) is the leading cause of death worldwide and can lead to the loss of cardiac function and heart failure. Reactive oxygen species (ROS) play a key role in the pathological progression of MI. The levels and effects of ROS are significantly different in three unique pathological stages of MI, and most antioxidants cannot make corresponding adjustments to eliminate ROS, which leads to a great compromise to treat MI with antioxidants. Herein, an innovative self‐sustaining antioxidant strategy is developed to treat MI with self‐sustaining selenium‐embedded nanoparticles (SSSe NPs). SSSe NPs possess unique self‐sustaining antioxidant effects at different pathological stages of MI. This strategy of on‐demand ROS elimination during different pathological stages demonstrated excellent MI treatment efficacy and effectively reversed heart failure to normal heart function. The therapeutic mechanism of SSSe NPs is intensively investigated through a series of experiments and mainly involved five critical aspects of myocardial repair: protecting mitochondria, reducing cardiomyocyte apoptosis and ferroptosis, reducing inflammation and fibrosis, and promoting angiogenesis. This strategy not only provides a promising treatment option for MI but also offers inspiration for other ischemic diseases.

## Graphical abstract

An innovative self‐sustaining antioxidant strategy to treat MI through self‐sustaining selenium‐embedded nanoparticles (SSSe NPs). SSSe NPs possess unique self‐sustaining antioxidant effects at different pathological stages of MI. SSSe NPs could repair the injured myocardium by protecting mitochondria, reducing cardiomyocyte apoptosis and ferroptosis, reducing inflammation and fibrosis, and promoting angiogenesis.

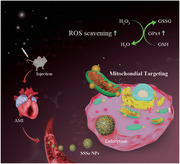

## 1 Introduction

Myocardial infarction (MI) is the leading cause of death worldwide, accounting for one‐seventh of all deaths.^[^ ^1^ ^]^ A variety of factors cause coronary occlusion and induce MI, which in turn leads to myocardial ischemia and hypoxia and ultimately to myocardial cell damage and necrosis.^[^ ^2^, ^3^ ^]^ Currently, the most effective treatment for MI is surgical revascularization (percutaneous coronary intervention and coronary artery bypass grafting) or drug thrombolysis within the first 24 h of MI.^[^ ^4^ ^]^ However, revascularization therapy can cause ischemia‐reperfusion (I/R) injury and a series of pathophysiological changes that lead to persistent cardiomyocyte damage. Moreover, revascularization is not applicable in many situations. Approximately 50% of patients with MI are asymptomatic, which is known as silent MI (SMI), and these patients often miss the therapeutic window for revascularization.^[^ ^5^ ^]^ In addition, damaged endothelial and thrombus fragments in the ischemic area detach from the primary lesion, leading to downstream microvascular occlusion. As a result, most MI patients have low reflow, and ≈30% of MI patients have no reflow after revascularization therapy, which causes persistent hypoxia in local myocardial tissue, resulting in poor patient outcomes.^[^ ^6^ ^]^ Numerous emerging therapeutic approaches have been developed to address this challenge, including stem cell therapy or cytokine therapy, such as vascular endothelial activity factor (VEGF) administration. However, stem cells are preferentially enriched in the lungs, and cytokines are systemically distributed, which not only limits efficacy but also causes significant side effects. In addition, the microenvironment, such as hypoxia and inflammation, greatly limits the survival of stem cells and the effect of cytokines on the MI site.^[^ ^7^, ^8^, ^9^ ^]^ Because of current treatment limitations, many MI patients have persistent progression leading to adverse left ventricular (LV) remodeling (characterized by LV cavity dilation and wall thinning) and ultimately death from heart failure.

The pathological progression from MI to LV remodeling is very complex and mainly involves myocardial cell apoptosis and death, inflammatory infiltration, fibrosis, and impaired neovascularization.^[^ ^10^ ^]^ LV remodeling consists of three consecutive stages: an early necrotic stage (necrosis of cardiomyocytes), an intermediate inflammatory stage (characterized by high levels of proinflammatory cytokines and the recruitment of immune cells), and an advanced fibrotic stage (collagen deposition). Reactive oxygen species (ROS) play a critical role in the development of this MI pathology.^[^ ^11^ ^]^ The first step is the ROS burst during the necrotic stage: cardiomyocytes are rich in mitochondria due to their high energy requirements. Blocking the mitochondrial respiratory chain leads to a burst of ROS during the necrotic stage due to insufficient blood and oxygen supply. Second, ROS persist at high levels during the inflammatory and fibrotic stages.^[^ ^12^ ^]^ Damage‐associated molecular patterns (DAMPs) from apoptotic cardiomyocytes induce inflammatory cell infiltration (e.g., macrophages), and these cells release inflammatory factors such as tumor necrosis factor‐*α* (TNF‐*α*) and transforming growth factor‐*β* (TGF‐*β*).^[^ ^13^ ^]^ TNF‐*α* induces continuous ROS production and further leads to cardiomyocyte apoptosis through a vicious cycle of ROS and inflammatory factors. Furthermore, TGF‐*β* causes LV remodeling through fibrosis. In addition, hypoxia can induce angiogenesis in MI tissue by promoting the secretion of VEGF, whereas ROS leads to insufficient angiogenesis by inhibiting VEGF production.^[^ ^14^ ^]^ Although endogenous antioxidant systems such as glutathione peroxidase 4 (GPx4) can effectively eliminate ROS in cardiomyocytes, the initial burst of ROS overwhelms and destroys the antioxidant system in cardiomyocytes.^[^ ^15^ ^]^ Correspondingly, MI tissue has a weak antioxidant capacity and cannot resist the damage caused by ROS. Therefore, antioxidant therapy for MI is very promising in theory. Thousands of antioxidant‐based treatments are being developed for the treatment of different diseases.^[^ ^16^, ^17^, ^18^, ^19^, ^20^ ^]^ However, antioxidant therapy has had only limited success for the treatment of MI, and there are still no antioxidants approved for the clinical treatment of MI. The fundamental reasons for this hindrance are as follows: the level of ROS is a steady‐state process in the body, and ROS are a very important biological signaling molecule required for important cellular functions. Excessive removal of ROS leads to dysfunction in cells. In addition, since myocardial repair is a slow process, it often requires long‐term or multiple administrations of common antioxidants, which can easily cause side effects and lead to significant compromises in treatment due to the toxicity of antioxidants and nonspecific distribution.

**Figure 1:**
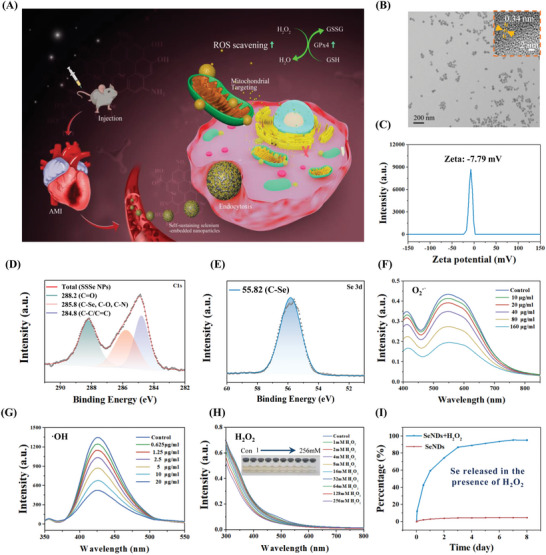
Characterization of SSSe NPs. A) Schematic illustration of the therapeutic effects of SSSe NPs. B) TEM image of SSSe NPs; scale bar: 100 nm. Insert: enlarged image of SSSe NPs; scale bar: 2 nm. C) Zeta potential distribution intensity of SSSe NPs. D) XPS spectra of C1s and E) Se3d in SSSe NPs. F) O_2_^·−^, G) ·OH and H) H_2_O_2_ scavenging ability of different concentrations of SSSe NPs. I) Release degree of Se in SSSe NPs with or without H_2_O_2_.

For the first time, we developed an innovative self‐sustaining antioxidant strategy to treat MI through self‐sustaining selenium‐embedded nanoparticles (SSSe NPs). SSSe NPs can exert antioxidant effects and mediate robust myocardial repair after intravenous administration during the necrosis stage of MI. A series of experiments demonstrated that SSSe NPs were specifically enriched in the MI site and in cardiomyocyte mitochondria. SSSe NPs effectively reduced cardiomyocyte apoptosis and ferroptosis by eliminating multiple ROS to maintain mitochondrial function at the MI site. More importantly, SSSe NPs possess unique self‐sustaining antioxidant effects. SSSe NPs can release Se for GPx4 biosynthesis to promote the repair of the endogenous antioxidant system while eliminating ROS in the MI site (**Figure** **1**A). Although SSSe NPs are depleted by ROS, the endogenous antioxidant GPx4 is transformed by SSSe NPs and can persistently scavenge ROS to effectively eliminate inflammation and fibrosis at the MI site. In addition, SSSe NPs can effectively induce MI tissue to produce VEGF, promote vascular repair, and restore blood flow in MI. This sustainable antioxidant strategy exhibited a strong ability to repair MI (effectively preventing LV remodeling and restoring cardiac function) with negligible side effects. This strategy provides not only a promising treatment for MI but also an inspiration for other repair diseases, such as cerebral infarction, acute kidney injury, and acute liver injury.

## 2 Experimental Section

### 2.1 Synthesis of SSSe NPs

Selenocystine (100 mg) was added to 60 ml of water, and then NaOH was added to dissolve selenocystine (≈pH 9) under ultrasound. Subsequently, 10 mg of dopamine hydrochloride was added to the selenocystine solution, followed by stirring for 24 h at 50 °C for 30 h. Finally, then centrifuged to remove precipitation. Finally, after removing the precipitate by centrifugation, the supernatant was dialyzed for 24 h (with three water changes), and lyophilized to obtain SSSe NPs.

### 2.2 Synthesis of FITC‐SSSe NPs

SSSe NPs (10 mg) were added to 20 ml of 0.1 mM tris(hydroxymethyl)aminomethane buffer solution, and FITC (5 mg) was added to react in the dark for 12 h. Subsequently, unreacted FITC was removed by dialysis for 24 h (with three water changes), and finally lyophilized to obtain FITC‐SSSe NPs.

### 2.3 MI Model

All animal procedures were performed according to protocols approved by Xiangya Hospital Central South University. An in vivo MI model was established in 8–12‐week‐old male C57BL/6 mice weighing 20–25 g based on our previous studies. Briefly, the mice were anesthetized with 2%–3% isoflurane inhalation in the inducing chamber and were continuously anesthetized with 1.5% isoflurane by endotracheal intubation. Ventilation indices were set as follows: tidal volume: 1.8 ml; ratio of inhalation to respiration: 5:4; and respiration rate: 120 times per min. After the mice were shaved and disinfected with alcohol, the skin was cut along the 3rd to 4th intercostal level of the left chest. The heart was exposed by bluntly separating the muscles layer by layer. Then, the left anterior descending artery (LCA) was ligated 2 mm below the left auricle using a 10–0 silk suture. Finally, the exposed chest was closed. One dose of buprenorphine (0.1 mg kg^−1^) was administered after the incision was closed. After being extubated, the mice were observed closely until they awakened from anesthesia. Mice in the sham group underwent the same procedures except that the LCA was not occluded. The mice in the control/treatment groups were randomized and received saline injection or SSSe NPs treatment (50 µg in 100 µl of saline) from the first day to the third day or NAC treatment (500 mg kg^−1^ d^−1^ for 14 days, P.O) after MI surgery.

### 2.4 Echocardiography

After being anesthetized, each individual mouse was placed on the heating blanket, and then, LV wall thickness and motility were evaluated by serial echocardiography (Mindray Inc., Nanjing, China) before MI surgery and at 1, 3, 7, 14, and 28 days after surgery. Mice in the sham group were investigated at similar time points. LVEF, LVFS, LVIDd, LVIDs, and LVPWs were obtained using M‐mode echocardiography. Using the formula *V* = [7.0/(2.4 + D)] × D^3^ (D is the internal diameter of the ventricle for that period), the LV end‐diastolic volume (LVEDV) and LV end‐systolic volume (LVESV), were calculated (in ml), and then the LVEF was calculated as follows: LVEF (%) = (LVEDV − LVESV)/LVEDV × 100%. All analyses were conducted by a single investigator who was blinded to the treatment groups.

### 2.5 Subcellular Localization

The subcellular localization of SSSe NPs was investigated by confocal microscopy. Briefly, 1 × 10^4^ H9C2 cells were plated on poly‐L‐lysine‐treated coverslips in 24‐well plates and incubated with or without 0.7 µg ml^−1^ SSSe NPs‐FITC for 4 h. Then, the cells were stained with MitoTracker Red (Yeasen, Shanghai, China) at 37 °C for 2 h. After being washed, the cells were stained with Hoechst at 37 °C for 10 min. A confocal laser scanning microscope (Zeiss, LSM900, Germany) was used for cellular imaging. The images were analyzed by using the Just Another Colocalization Plugin (JACoP) and ImageJ software (NIH, MD, US).

### 2.6 TEM

Myocardial tissues were harvested, and tissue blocks less than 1 cubic millimeter in size were immediately fixed in fresh TEM fixation solution for at least 2 h. Then, the tissues were rinsed with 0.1 M PBS 3 times (15 min each) and fixed with a 1% osmium acid mixture for 2–3 h. After fixation, the tissues were rinsed with 0.1 M PBS again (3 times, 15 min per wash). After gradient dehydration, the tissues were embedded and solidified. The tissue blocks were cut to a thickness of 60–80 nm with an ultratome followed by 3% uranyl acetate‐lead citrate double‐staining. Then, the tissues were rinsed 3 times in ultrapure water. Finally, the tissues were observed using a Jeol 1200EX transmission electron microscope. TEM analysis of H9C2 cells was similar to that of tissues.

### 2.7 HE Staining

Heart, liver, lung, spleen, and kidney specimens were fixed with formalin for 24 h, dehydrated by gradient ethanol, and vitrified with xylene. The specimens were embedded in paraffin and sliced into 5 µm sections. After being baked at 65 °C for 1 h, the sections were routinely dewaxed and hydrated. Staining was performed as follows: hematoxylin staining for 5 min, incubation in a hydrochloric acid alcohol solution for 2–3 s, incubation in blue‐return solution for 3 s, eosin staining for 1–3 min, and rinsing with water. After being dehydrated, the sections were sealed with neutral resin. Finally, the sections were observed and photographed under a microscope.

### 2.8 Masson Staining

A Masson Three‐Color Kit (Servicebio, G1006) was used for Masson staining. Briefly, after deparaffinization and rehydration, 5 µm‐thick paraffin sections were incubated in 2.5% potassium dichromate mordant at room temperature overnight and then at 65 °C for 30 min. After the slices were washed in PBS for 30 s, they were immersed in Weigert iron hematoxylin dye for 1 min. Then, the slices were placed in 1% chlorohydric acid in ethanol for 1 min. The slices were dipped in Richun red acid magenta for 6 min, in 1% phosphomolybdic acid solution for 1 min, and in 2.5% aniline blue solution for 30 s. The slices were washed with acetic acid 3 times for 8 s, dehydrated with ethanol for 5 s, 10 s, and 15 s, dehydrated with n‐butanol for 30 s and 2 min, and hyalinized with xylene twice for 5 min. Finally, the neutral resin was used to seal the slices.

### 2.9 Perl's Staining

After deparaffinization and rehydration, the paraffin sections were stained with a mixture of 2% potassium ferrohydride (Alladin, P112418) and 2% hydrochloric acid (Sinopharm Chemical Reagent, 10 011 008) in equal proportions for 30 min. Then, the sections were washed twice with distilled water and stained with DAB (TCI. D0078) for ≈5 min and washed again. Hematoxylin dye (Servicebio, G1004) staining was performed for 1 min to stain the nuclei, and differentiation was performed with a hydrochloric acid aqueous solution, followed by washing with tap water, immersion in aqueous ammonia solution, and washing with tap water again. Finally, the sections were dehydrated three times with ethanol for 5 min and sealed with neutral resin. Three sections were viewed and photographed under a microscope.

### 2.10 Live Cell Imaging to Determine Mitochondrial Superoxide (MitoSOX) Levels

H9C2 cells were placed on poly‐L‐lysine‐treated coverslips in 24‐well plates and incubated with 1 ml of DMEM with 10% FBS. After the cells had grown to the appropriate density, they were stimulated with 400 µM H_2_O_2_ or 100 µM erastin for 6 h with or without PBS or SSSe NPs (10 ng ml^−1^). After the culture medium was removed at the end of the treatment period, the cells were stained with 5 µM MitoSOX (Yeasen, Shanghai, China) for 10 min at 37 °C in an incubator in the dark. Then, the cells were washed 3 times with 37 °C PBS and observed under a fluorescence microscope (Leica DM3000 LED).

### 2.11 Measurement of MMP

H9C2 cells were cultured in 6‐well plates and were treated as described. After the culture medium was removed, the cells were gently washed with PBS, and 1 ml of cell culture medium combined with 1 ml of JC‐1 staining working solution (Beyotime, Shanghai, China) was added to each well. Then, the cells were incubated for 15 min in a 37 °C cell incubator and gently washed twice with wash buffer. The fluorescence intensity was measured using a fluorescence microscope (Leica DM3000 LED).

### 2.12 Detection of ATP Generation

After the culture medium was aspirated, H9C2 cells were lysed with 200 µl per well lysis solution from the ATP detection kit (Beyotime, Shanghai, China). Then, the lysate was centrifuged at 4 °C for 5 min at 12 000 g, and the supernatant was collected for subsequent assays. A volume of 20 µL of supernatant was mixed with 100 µL of ATP detection working solution. ATP generation was measured at 562 nm with a luminometer manufactured by Bio‐Tek (America).

### 2.13 Mitochondrial DNA (mtDNA) Copy Number

mtDNA copy number was estimated by quantitative real‐time PCR to determine the protective effect of SSSe NPs on mitochondrial function under oxidative stress and ferroptosis conditions. DNA was extracted from the cells with a DNeasy Blood and Tissue Kit (Qiagen, Hilten, Germany). Mitochondrially encoded Cytochrome C Oxidase II (MT‐CO2) was measured using the following primers: Forward 5’‐CCT CCC ATT CAT TAT CGC CGC CCT TGC‐3’ and Reverse 5’‐GTC TGG GTC TCC TAG TAG GTC TGG GAA‐3’. The nuclear gene ACTB (*β*‐actin) was measured using the following primers: Forward 5’‐GGA GAT TAC TGC CCT GGC TCC TA‐3’ and Reverse 5’‐GAC TCA TCG TAC TCC TGC TTG CTG‐3’. The assay was performed in a total reaction volume of 20 µl containing 10 µl of SYBR Premix Ex Taq, 6.8 µl of ddH2O, 0.4 µl of each primer (10 µM), 0.4 µl of ROX Reference DyeII (50x) and 2 µl of DNA template. The reaction was performed at 95 °C for 30 s, followed by 40 cycles of 5 s at 95 °C and 34 s at 65 °C, 95 °C for 15 s, 1 min at 60 °C, and 15 s at 95 °C. Each sample was assayed in triplicate, and the relative mtDNA copy number was calculated with ΔΔCt and normalized to ACTB.

### 2.14 Fluorescent Imaging of Tissue

SSSe NPs or normal saline were administered to MI or sham mice. Some hearts were harvested and observed under a stereo fluorescence microscope (Leica, M205FCA). Other hearts were frozen at −80°C and then frozen at −20 °C for 30 min. The specimens were embedded with OCT and sliced into 10 µm sections. After being washed with PBS three times for 5 min, the slices were incubated with 0.05% Triton for 5 min. Then, the slices were washed again, sealed with DAPI‐Fluoromount‐G, and photographed by a fluorescence microscope.

### 2.15 Assessment of ROS Production

Changes in intracellular ROS levels were determined with the DCFH‐DA probe. H9C2 cells were seeded in 96‐well plates and treated as previously described. Then, the cells were stained with 10 µM DCFH‐DA at 37 °C for 20 min, gently washed three times, and photographed by a fluorescence microscope. For ROS staining of tissue, 7 days after AMI surgery, the hearts were harvested and immediately prepared as frozen sections. The sections were incubated with washing buffer at room temperature for 10 min, incubated with the DCFH‐DA probe at 37 °C for 1 h, and then photographed by a fluorescence microscope.

### 2.16 Evaluation of GSH‐PX Activity

Hearts were harvested 3 days after MI surgery. After tissue homogenization, 9 volumes of saline were added to prepare a 10% tissue homogenate. All samples were centrifuged at 3000 rpm per min for 10 min at 4 °C, and the supernatant was collected to measure the protein concentration. After the sample was added to the centrifuge tube, GSH and the reagent application solution were added sequentially for the enzymatic reaction (37 °C). The mixture was centrifuged at 3000 rpm per min for 10 min at room temperature, and the supernatant was used for the color development reaction, which was measured at 412 nm with a luminometer.

### 2.17 TUNEL Staining

The mice were killed after surgery. The tissues were covered a TUNEL working solution for 1 h, and the nucleus was stained with DAPI. Images were acquired by a fluorescence microscope (Leica DMC6200).

### 2.18 Protein Preparation and Western blotting

Mouse heart tissue/cells were lysed for 30 min with RIPA buffer (Beyotime, China) supplemented with protease and phosphatase inhibitors (Beyotime, China) on ice. The lysate was centrifuged at 12 000 rpm for 15 min at 4 °C, and the protein concentrations of the supernatant were analyzed with a BCA kit (Beyotime, China). Each sample (20 µg of protein) was separated by SDS‒PAGE gels and transferred to PVDF membranes. The membranes were immersed in 5% milk in TBST buffer for 1 h at room temperature, followed by incubation with primary antibodies against *β*‐actin (CST, 1:1000), bax (Abcam, 1:1000), caspase3 (CST, 1:1000), caspase 8 (CST, 1:1000), Cyt‐c (Proteintech, 1:1000), GPx‐4 (Abcam, 1:1000), Bcl‐2 (Affinity, 1:1000), TGF‐*β* (CST, 1:1000), COX‐2 (Affinity, 1:1000), VEGF A (CST, 1:1000), FAK (CST, 1:1000), ERK1/2 (CST, 1:1000), p38 (CST, 1:1000), transferrin receptor (Abcam, 1:1000), and ferroportin (Novus, 1:1000) at 4 °C overnight. Then, the membranes were washed three times with TBST, followed by incubation with secondary antibodies (1:10 000) for 1 h at room temperature. The bands were visualized by using a gel documentation system (Bio‐Rad, USA) and quantified by ImageJ software.

### 2.19 Phosphorylation Analysis

Phos‐Tag SDS‐PAGE was used to determine the phosphorylation of FAK, ERK1/2, and P38. Phosbind acrylamide (ApexBio, USA) and MnCl_2_ were added to the SDS‐PAGE gels. The proteins were separated by Phos‐Tag SDS‐PAGE as mentioned previously, and the gel was incubated in a transfer solution containing ethylene diamine tetraacetic acid (EDTA) to remove Mn^2+^. Then, the proteins were transferred to PVDF membranes (Millipore, USA). The membranes were blocked and incubated with primary antibodies (FAK, CST, 1:1000; ERK1/2, CST, 1:1000; p38, CST, 1:1000) overnight, followed by incubation with secondary antibodies as described in the normal Western blotting protocol. The bands were also visualized by using a gel documentation system (Bio‐Rad, USA) and quantified by ImageJ software.

### 2.20 Immunohistochemistry

After being treated, the cells were fixed with 4% paraformaldehyde for 20 min and blocked with 5% bovine serum albumin (BSA; Beyotime, China) in PBS at room temperature for 1 h. Then, the cells were incubated in a primary antibody reagent containing COX‐2 (CST, 1:200) and Tubulin (Abcam, 1:1000) overnight at 4 °C, followed by incubation in secondary antibodies for 1 h at room temperature. The samples were then stained with DAPI and imaged using a fluorescence microscope (Leica, Germany). The heart tissues were fixed for 24 h at room temperature in 4% paraformaldehyde, followed by dehydration, embedding, and sectioning. After being baked for 1 h at 65 °C for hydration and antigen repair, the cells were blocked with 10% donkey serum and 0.3% Triton X‐100 in PBS at room temperature for 2 h. Then, the sections were incubated in a primary antibody reagent overnight at 4 °C, followed by incubation in secondary antibodies for 1 h at room temperature. After being stained with DAPI, the cells were imaged with a fluorescence microscope. The primary antibodies used included *α*‐SMA (Sigma, c6198), CD68 (Santa Cruz, sc‐59103), CD86 (BioLegend, 105 102), and CD206 (CST, 24 595).

### 2.21 RNA Extraction and Real‐Time PCR

Total RNA was extracted by using TRIzol reagent. RNA quantity and integrity were assessed on a NanoDrop 2000 instrument (Thermo Fisher Scientific, USA). The RNA was reverse transcribed into cDNA using an RT reagent kit from Takara (Japan) and assayed by real‐time PCR. qPCR was performed in a total reaction volume of 20 µl containing 10 µl of SYBR Premix Ex Taq, 6.8 µl of ddH2O, 0.4 µl of each primer (10 µM), 0.4 µl of ROX Reference DyeII (50x) and 2 µl of cDNA template. The reaction was performed at 95 °C for 30 s, followed by 40 cycles of 5 s at 95 °C and 34 s at 65 °C, 95 °C for 15 s, 1 min at 60 °C, and 15 s at 95 °C. The relative changes in gene expression were estimated and normalized to GAPDH by using the 2−∆∆*CT* method.

**Table:** 

Gene	Primer Sequence
IL‐1*β*	Forward ATGAGAGCA TCCAGCTTCAA
	Reverse TGAAGGAAAAGAAGGTGCTC
IL‐6	Forward CTGCAAGAGACTTCCATCCAG
	Reverse AGTGGTATAGACAGGTCTGTTGG
IL‐10	Forward CTTACTGACTGGCATGAGGATCA
	Reverse GCAGCTCTAGGAGCATGTGG
TNF‐*α*	Forward CCTGTAGCCCACGTCGTAG
	Reverse GGGAGTAGACAAGGTACAACCC
GAPDH	Forward AGGTCGGTGTGAACGGATTTG
	Reverse GGGGTCGTTGATGGCAACA

### 2.22 Cell Counting kit‐8 (CCK‐8)

A CCK‐8 assay (Yiyuan Biotechnologies, Guangzhou, China) was used to measure the proliferation of H9C2 cells and HUVECs. After being cultured overnight in a 96‐well plate (5000 cells per well, 100 µL of medium per well), the cells were treated with PBS or different concentrations of SSSe NPs for 12 h. Then, the CCK‐8 reagent (10:1) was added and incubated at 37 °C for 4 h. Finally, the absorbance of each well was measured at 450 nm using a microplate reader (BioTek, USA). A CCK‐8 assay was also used to measure the ability of selenium nanoparticles to rescue HUVEC viability after H_2_O_2_ injury.

### 2.23 In Vitro Angiogenesis Assay

Matrigel was thawed at 4 °C overnight. Tube formation assays were performed in a 96‐well plate, and 70 µl of Matrigel was added to each well and polymerized for 20 min at 37 °C. HUVECs were treated with PBS or different concentrations of SSSe NPs for 12 h with or without H_2_O_2_ pretreatment, and then, the cells were trypsinized and counted. A total of 5000 cells per well were seeded in the Matrigel in 100 µl of ECM. After 4 h of incubation at 37 °C, the tubes were imaged using a fluorescence microscope. Tube formation was analyzed by counting the nodes and measuring the total tube length using ImageJ.

### 2.24 Migration Assay

HUVECs were plated on 6‐well plates until they reached 90% confluence. The cells were pretreated with H_2_O_2_ + SSSe NPs or H_2_O_2_ + PBS for 6 h at 37 °C. HUVECs were scratched in a line shape using a 200 µl tip. After being cultured for 0 h, 6 h, 12 h, and 24 h, the wound width was recorded with a phase‐contrast microscope (Leica DM3000 LED). ImageJ software was used to measure the distance between the sides of the scratch.

### 2.25 Hemolysis Assay

Whole blood was collected from mice in test tubes by extracting orbital blood under anesthesia. Blood (0.5 ml) was mixed with 5 ml of PBS and then centrifuged at 3000 rpm for 10 min at 4 °C. The supernatant was discarded, and the erythrocytes were retained. A 2% erythrocyte suspension was prepared by diluting the erythrocytes with PBS solution. SSSe NPs were diluted with PBS to 0.1, 1, 10, 100, and 1000 µg ml^−1^.

PBS as a negative control was added to the 2% erythrocyte suspension. A positive control was also prepared by mixing water with an equal volume of 2% RBC suspension to produce 100% hemolysis. Then, 0.5 ml of SSSe NPs and 0.5 ml of the 2% erythrocyte suspension were mixed, and the samples were incubated at room temperature for 3 h and then centrifuged at 1000 rpm for 3 min. Then, 100 µl of the supernatant was added to a 96‐well plate, and the OD 570 nm was measured.

### 2.26 Triphenyl Tetrazolium Chloride (TTC) Staining

After being euthanized, the mice were perfused with PBS to remove the blood from the cardiac chambers. Then, the whole heart was removed and washed in PBS. After being frozen at −20 °C for 2 h, the heart was sliced vertically and along the long axis from the apex cordis to the basis cordis (4–5 slices, 1 mm each). The tissue slices were placed onto a petri dish, and 1% TTC solution (Sigma, T8877) was added. The slices were incubated at 37 °C in the dark for 10 min. Finally, the tissue was fixed for 2 h with 4% PFA, and photographs were taken.

### 2.27 Determination of MDA Concentrations

MDA was used to determine the degradation products of lipid peroxidation. After tissue homogenization, all samples were centrifuged at 12000 ×g for 10 min at 4 °C. The supernatant was collected for subsequent analysis. The protein concentration of the supernatant was determined using a BCA assay (Beyotime, China). One hundred microliters of supernatant was mixed with 200 µl of freshly prepared MDA assay working solution, and then the mixture was incubated in a 100°C water bath for 15 min and slowly cooled to room temperature. The mixture was centrifuged at 1000 ×g for 10 min at room temperature. Finally, the absorbance of the supernatant was measured at 532 nm using a microplate reader (BioTek, USA).

For routine blood tests and biochemical indices, orbital blood was extracted under anesthesia within blood collection tubes containing the anticoagulant EDTA or normal blood collection tubes. Then, the blood samples were sent to the Laboratory Medicine Department of Xiangya Hospital Central South University for routine blood tests (white blood cells, red blood cells, hemoglobin, platelets, neutrophils, lymphocytes, and monocytes) and biochemical indices (total protein, albumin, globulin, ratio of albumin to globulin, glutamic pyruvic transaminase, glutamic oxalacetic transaminase, uric acid, creatinine, bilirubin, and urea).

### 2.28 Evaluation of the Selenium Content

The selenium content of mouse heart tissue was quantified using ICP with weight normalization. Hearts were harvested at 1, 3, 7, 14, and 28 days after surgery and saline/SSSe NPs injection.

### 2.29 Transcriptome Sequencing

The mice were euthanized on day 3 after MI surgery, the infarction border zone of the heart was harvested, and RNA was extracted using a standard TRIzol protocol. Paired‐end sequencing (2×100 bp) was carried out with a BGI‐500 instrument (BGI) to obtain at least 20 million reads for each sample. The sequence data were processed and mapped to the human reference genome (hg19) using Bowtie2. Gene expression was quantified as Fragments‐Per‐Kilobase of transcript per million mapped fragments (FPKM) using RNA‐Seq by Expectation‐Maximization (RSEM).

### 2.30 Analysis of DEGs

After data standardization and normalization of the transcriptome sequencing data using the Normalize Between Arrays function in the “limma” R package, principal component analysis (PCA) was conducted by using the “factoextra” R package. The DEGs between MI‐NS and MI+SSSe NPs were analyzed by using the “limma” R package. The DEGs were screened with the following criteria: log2 (FoldChange)> = 2.15 and *P* < 0.05. A volcano plot and a heatmap plot were created by using the R software ggplot2 package and “ComplexHeatmap” to show significantly dysregulated genes, respectively.

### 2.31 Enrichment Analysis

The “clusterProfiler” package was used to enrich the biological processes (BPs) of GO of DEGs to identify the significantly differential functions between MI‐NS and MI+SSSe NPs.

### 2.32 Statistical Analysis

The relatively quantitative data, such as fluorescence intensity, protein expression, and mRNA expression, the value or mean value of control was normalized as 1. All data are presented as the mean ± SD. Sample size (*n*) for each experiment was described in the figure legend. Comparisons between two groups were performed with an unpaired two‐tailed Student's *t*‐test. Comparisons among more than two groups were performed using one‐way ANOVA followed by a post hoc Bonferroni test. Differences were considered statistically significant when *P* < 0.05. Statistical analysis was performed using Prism 8 software (GraphPad, Inc., San Diego, CA, USA).

## 3 Results and Discussion

### 3.1 Synthesis and Characterization of SSSe NPs

SSSe NPs were prepared by pyrolysis and carbonization of selenocysteine under alkaline conditions (Figure S1, Supporting Information). The SSSe NPs had a small size of 20–40 nm, excellent monodispersity (Figure 1B, Figure S2A, Supporting Information) and a negative charge (Figure 1C) by transmission electron microscopy (TEM) and zeta potential, respectively. These properties enabled SSSe NPs to be efficiently enriched in MI tissue through the blood circulation system because the blood vessels at the MI site have damaged interendothelial links^[^ ^21^ ^]^ that facilitate the penetration of SSSe NPs into the injured MI. The X‐ray diffraction (XRD) diffraction peak of the (002) crystal plane was located at 26° (Figure S2B, Supporting Information), indicating that SSSe NPs had a typical graphite sheet structure and that the thickness of the sheet was ≈0.34 nm. The nanoparticles had a graphite sheet structure with a spacing of 0.34 nm according to high‐resolution TEM (Figure 1B, insert), was consistent with the XRD measurements. The elemental composition and chemical properties of SSSe NPs were further analyzed by X‐ray photoelectron spectroscopy (XPS). SSSe NPs contained Se, C, N, and O elements, of which C was the main component (53.1%) (Figure S3, Supporting Information). SSSe NPs had many C≐O and C–O bonds through the C1s XPS fine peak (Figure 1D), indicating that SSSe NPs were abundant in carboxyl groups and phenolic hydroxyl groups, which determined the negative charge of SSSe NPs. More importantly, the Se content was as high as 13.46% (Figure S3, Supporting Information), and Se was embedded into the carbon framework through C–Se covalent bonds by C1s and Se XPS fine peaks (Figure 1D,E), which ensured that the SSSe NPs remained stable under normal physiological conditions. In addition, SSSe NPs were rich in NH or NH_2_ groups through the fine XPS peak of N1s (Figure S4, Supporting Information). Therefore, SSSe NPs could easily be further functionalized by amide bonds. SSSe NPs had strong scavenging effects on O_2_.^−^ (mainly derived from mitochondria and NOX2 in inflammatory cells) and OH^.^ (Mainly causing ferroptosis in cardiomyocytes) (Figure 1F‐G). Unlike O_2_.^−^ and OH^.^, which had very short lifetimes and diffusion distances, H_2_O_2_ could exist stably and cause oxidative stress damage in the MI site.^[^ ^22^ ^]^ H_2_O_2_ could be eliminated by SSSe NPs and decrease the visible light absorption spectrum of SSSe NPs (Figure 1H). H_2_O_2_ could etch the C–Se bonds in SSSe NPs and lead to the slow release (≈4 days) of soluble selenate ions from SSSe NPs into the solution (Figure 1I). In a normal environment, SSSe NPs remained stable, and no soluble Se was released because Se was embedded in the C skeleton in SSSe NPs and did not react with O_2_ in the aqueous solution. The strategy of slowly releasing soluble Se was not only beneficial for Se to be converted into GPx4 by myocardial tissue but could also avoid tissue damage caused by excessive selenate ions. In addition, SSSe NPs had excellent biocompatibility. As shown in Figure S5, Supporting Information, SSSe NPs did not cause any toxicity to two key cell types (cardiomyocytes and endothelial cells) involved in MI repair even at high concentrations up to 10 000 ng ml^−1^ (Figure S5, Supporting Information). SSSe NPs also did not cause any hemolysis even at very high concentrations (Figure S6, Supporting Information) because of their slightly negative surface charge.

### 3.2 Distribution of SSSe NPs In Vivo

**Figure 2:**
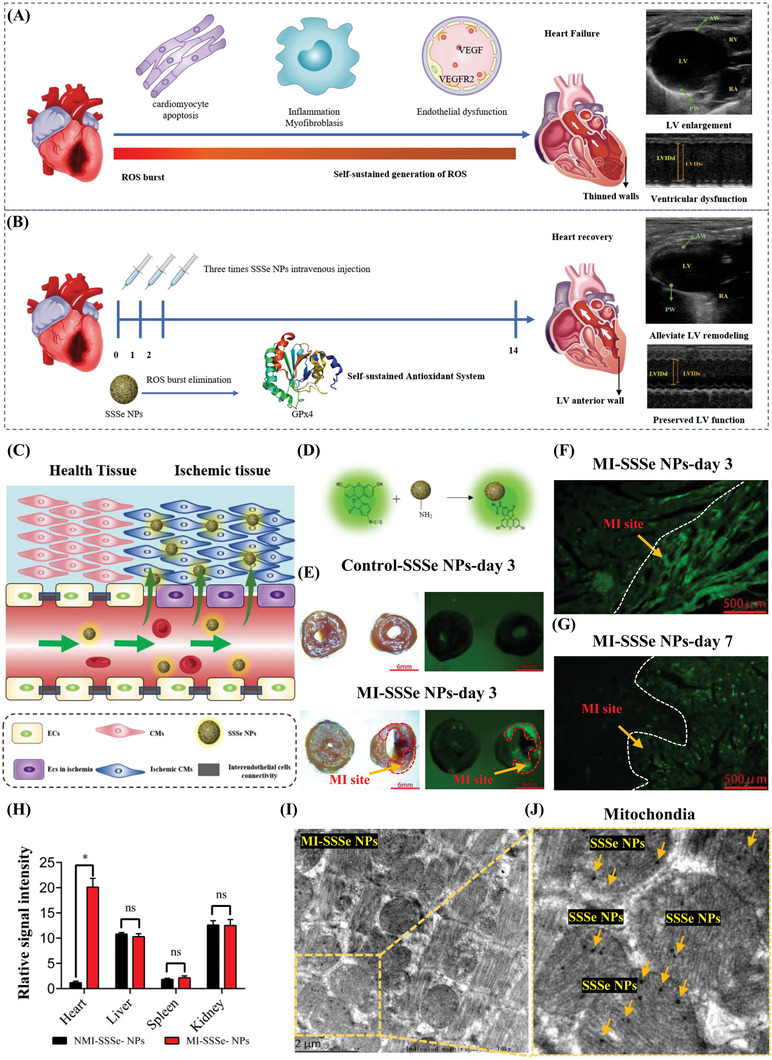
Distribution of SSSe NPs in vivo A) Schematic illustration of the fate of the heart in mice with MI. B) A schematic showing SSSe NP treatment of mice with MI. C) Through leaky myocardial circulation, SSSe NPs entered the infarct tissue. D) FITC‐loaded SSSe NPs. E) Representative ex vivo fluorescence imaging of mouse hearts following tail vein injection of SSSe NP‐FITC into control and MI mice. F) Image of myocardial tissues showing FITC‐loaded SSSe NPs entering the MI site on day 3 after MI injury. G) Image of myocardial tissues showing FITC‐loaded SSSe NPs entering the MI site on day 7 after MI injury. H) Quantification of FITC‐labeled SSSe NPs in different organs on day 1 after MI surgery. I–J) TEM images showing SSSe NPs (yellow arrow) loaded on mitochondria in myocardial tissue. All data are presented as the mean ± S.D. (*n* = 3). Statistical significance was calculated by an unpaired two‐tailed Student's *t*‐test. ns *P* > 0.05, * *P* < 0.05.

SSSe NP therapy was only administered once daily during the initial ROS burst (72 h) of MI (**Figure** **2**A,B), and subsequent administration was not required due to the self‐sustained antioxidant properties of SSSe NPs. As shown in Figure 2C, MI can damage endothelial cell junctions and the vascular basement membrane, and SSSe NPs were specifically enriched at the site of MI after intravenous injection. To analyze the distribution of SSSe NPs in vivo, we modified SSSe NPs with fluorescein isothiocyanate (FITC, a strong fluorescent group) through NH_2_ on SSSe NPs (Figure 2D, Figure S7, Supporting Information) and tracked the distribution of SSSe NPs in vivo by fluorescence imaging. The blood half‐life of SSSe NPs was 10–11 h by detection of Se content in blood with ICP‐MS, (Figure S9A, Supporting Information). We observed that fluorescence of SSSe NPs‐FITC in urinary bladder levels peaked within 12 h and then gently fell as the drug was voided from the bladder (Figure S9B, Supporting Information). In normal mice, SSSe NPs were mainly excreted through the kidneys due to their small and flexible lamellar structure. Although their size was slightly larger than that of the glomerular filtration barrier, the flexible 2D structure was folded and rolled to pass through the glomerular filtration barriers.^[^ ^23^ ^]^ Furthermore, SSSe NPs were not distributed in the healthy heart and were trapped in the liver and spleen by the reticuloendothelial system (Figure 2E, Figure S8, Supporting Information). Interestingly, SSSe NPs were highly efficient and specific for MI tissue (Figure 2E‐H, Figure S10, Supporting Information). Moreover, SSSe NPs remained at the MI site for a long time. As shown in Figure 2G, SSSe NPs partially persisted, and SSSe NPs were reduced by ≈29.9% at the MI site on day 7. Higher Se levels in the SSSe NP groups were maintained in MI tissue relative to those in the untreated groups for a long time, as shown by inductively coupled plasma‒mass spectrometry (ICP‒MS) (Figure S11, Supporting Information). Notably, the selenium content in SSSe NP‐treated MI tissue was reduced by only ≈8.2% on day 7, which was significantly lower than the reduction in SSSe NPs (29.9%). These results fully proved that the soluble Se released by SSSe NPs was efficiently bioavailable for GPx4 synthesis and remained in MI tissue. We further observed the distribution of SSSe NPs in MI tissue by biological TEM. As shown in Figure 2I, SSSe NPs were widely distributed in MI tissue, which was consistent with the fluorescence imaging results. Interestingly, SSSe NPs were also distributed in cardiomyocyte mitochondria (Figure 2J) at the MI site. Although SSSe NPs were not modified with specific mitochondrial targeting groups, SSSe NPs still passively targeted cardiomyocyte mitochondria because the myocardium had the most abundant mitochondrial density in the human body due to extremely high energy demands.

### 3.3 Therapeutic Effects of SSSe NPs

**Figure 3:**
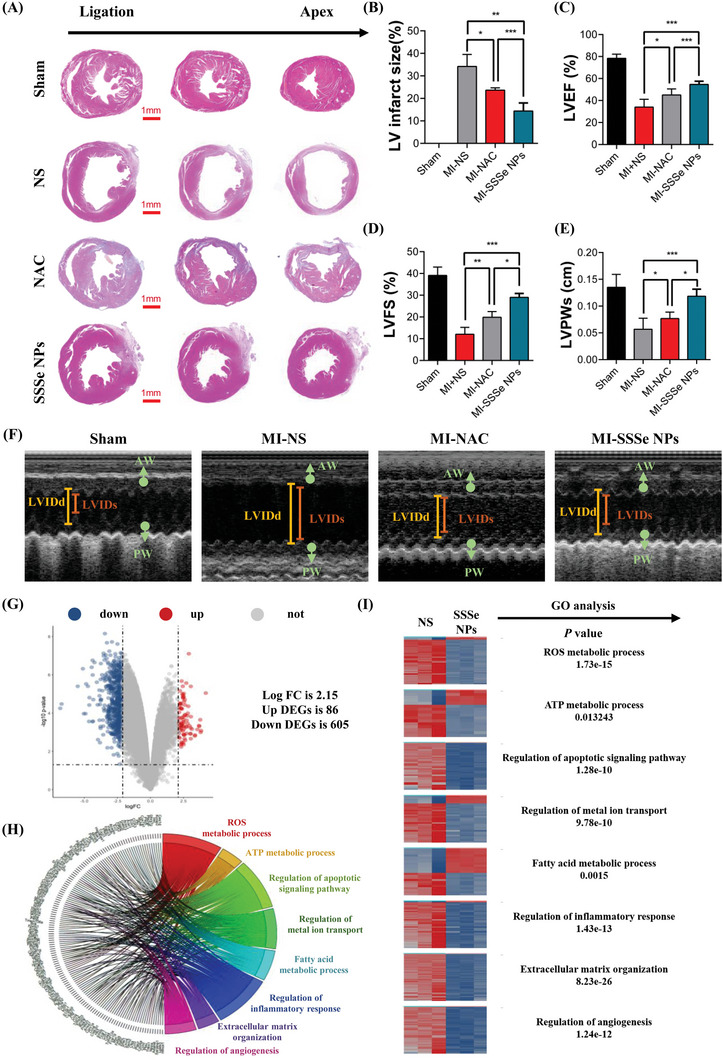
SSSe NPs administration improves cardiac dysfunction after MI. A) HE staining showing a smaller infarcted area in the myocardium in the SSSe NP‐treated groups than in the NS/NAC groups on day 14 after acute MI. B) Quantification analysis of the infarct/healthy area. All data are presented as the mean ± S.D. (*n* = 6). C–E) Measurement of cardiac function by echocardiography, including LVEF, LVFS, and LVPWs on day 14 after MI. F) Representative M‐mode images of echocardiography in each treatment group on day 14 after MI. AW: anterior wall; PW: posterior wall. All data are presented as the mean ± S.D. (*n* = 9). G) Volcano plots showing differentially expressed genes (DEGs) with logFC > 2.15 and FDR *P* < 0.05 in MI tissue treated with saline or SSSe NPs. The blue points indicate downregulated DEGs. The red points indicate upregulated DEGs. H) Chord diagram showing overrepresented Gene Ontology (GO) terms for the genes that were differentially expressed during hyperopia induction. In the chord diagrams, overrepresented GO terms are shown on the right, and genes contributing to this overrepresentation are shown on the left. I) Heatmaps showing DEGs based on GO analysis of RNA‐seq data of cardiac tissue of MI mice treated with saline or SSSe NPs for 3 days (*n* = 3). Statistical significance was calculated via one‐way ANOVA followed by a post hoc Bonferroni test. Ns *P* > 0.05, * *P* < 0.05, ** *P* < 0.01; *** *P* < 0.001.

First, we optimized the method of SSSe NP administration for treating MI. One intravenous injection of SSSe NPs on the first day of MI showed an excellent strong myocardial repair effect after 14 days. During the first three days after MI, once‐daily administration of SSSe NPs further reduced the size of the MI, as shown by hematoxylin and eosin (HE) staining (Figure S12B, Supporting Information). Therefore, we used once‐daily administration during the early stage of MI (3 days), and the treatment effect was evaluated after 14 days in the follow‐up experiments if not specifically mentioned. Subsequently, we selected the clinical antioxidant N‐acetyl‐L‐cysteine (NAC) as a control.^[^ ^24^ ^]^ As shown in **Figure** **3**B, SSSe NPs significantly reduced the MI area relative to that in the untreated group (14.41 ± 1.463% vs 34.20 ± 5.341%) (Figure 3B), while NAC slightly reduced the MI area, as shown by HE staining (23.65 ± 1.039%). The superior therapeutic effect of SSSe NPs was further demonstrated by TCC staining (Figure S13, Supporting Information). LV thickness was also significantly restored by SSSe NPs treatment relative to that in the NAC and untreated groups (Figure 3E).

We further assessed cardiac function in vivo by small animal ultrasound. LV ejection fraction (LVEF) is the gold standard for evaluating cardiac function.^[^ ^25^ ^]^ Clinically, an LVEF of less than 50% is regarded as heart failure, and less than 30% is regarded as serious abnormal heart function.^[^ ^26^ ^]^ LVEF was calculated by the Teichholz formula by measuring the LV internal diameter in diastole (LVIDd) and the LV internal diameter at end‐systole (LVIDs). As shown in Figure 3D, NAC treatment ameliorated cardiac dysfunction relative to that in the untreated group (32% vs 44%), but failed to reverse the severe heart failure caused by MI. In strong contrast, SSSe NPs restored the LVEF to 55% of the normal range (Figure 3D), indicating that SSSe NPs effectively reversed heart failure and restored heart function. Other cardiac function indicators, such as LV fractional shortening (LVFS) (Figure 3F), LVIDs (Figure S15B, Supporting Information), and LVIDd (Figure S15C, Supporting Information), further indicated that SSSe NPs effectively restored myocardial function. Moreover, the dynamic process of treating cardiac function with SSSe NPs was further studied by assessing cardiac function at different times, including 1 day (Figure S16, Supporting Information), 3 days (Figure S17, Supporting Information), 7 days (Figure S18, Supporting Information), 14 days (Figure S15, Supporting Information), and 28 days (Figure S19, Supporting Information) after MI. Because of this sustainable antioxidant strategy, SSSe NPs effectively protected the myocardium and maintained myocardial function throughout the pathological progression of MI (Figure S20, Supporting Information). SSSe NPs effectively restored myocardial function in the early stage of MI (Figures S16–S17, Supporting Information) and further restored cardiac function in the inflammation and fibrosis stages (Figures S15, S18–S19, Supporting Information). In addition, the recovery of myocardial function was further demonstrated by HE staining at these time points (Figure S21, Supporting Information).

To further understand the mechanism by which SSSe NPs treat MI, treated and untreated MI tissues were subjected to RNA sequencing and analyzed by the Illumina HiSeq platform on the third day after MI (Figure S22, Supporting Information). Fragments‐per‐kilobase of transcript per million fragments mapped (FPKM) was used as an indicator of the level of transcript or gene expression, and a fold change of 2.15 was used to identify DEGs. Compared with the control group, 86 genes were upregulated and 605 genes were downregulated in the SSSe NPs treatment group (Figure 3G). GO enrichment analysis of the DEGs in the two groups of MI tissues showed that DEGs associated with apoptosis, inflammation, and extracellular matrix remodeling were activated during MI but were downregulated after SSSe NPs treatment compared with NS. Therefore, SSSe NPs treatment was closely associated with mitochondrial function (ATP metabolic process and ROS metabolic process), apoptosis (ATP metabolic process and regulation of apoptotic signaling pathway), ferroptosis (regulation of metal ion transport and fatty acid metabolic process), inflammation (regulation of inflammatory response), fibrosis (extracellular matrix remodeling) and angiogenesis (regulation of angiogenesis) (Figure 3H,I).

### 3.4 SSSe NPs Protect Mitochondria

**Figure 4:**
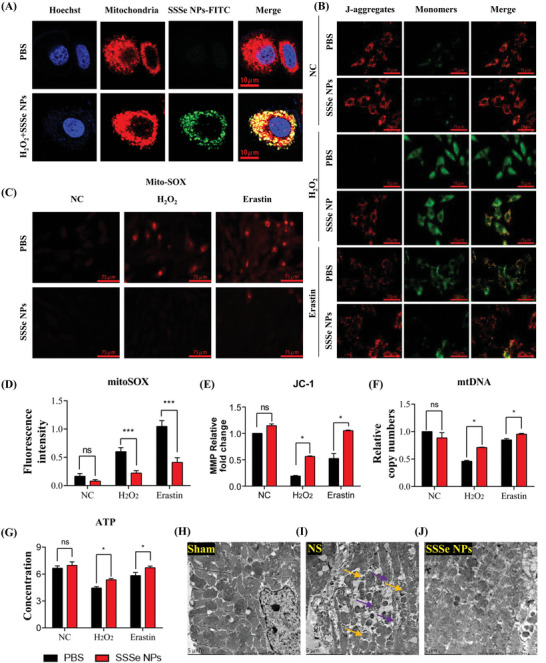
SSSe NPs treatment alleviates stress‐induced mitochondrial injury in myocardial cells. A) Immunostaining of mitochondria (red MitoTracker) and nuclei (blue) in H9C2 cells treated with or without FITC‐labeled SSSe NPs (green). B) Measurement of mitochondrial membrane potential (MMP) following the treatment of PBS/SSSe NPs with H_2_O_2_ or erastin and JC‐1 staining. The JC‐1 monomer accumulates in mitochondria, indicating that the membrane potential decreased. C‐D) H9C2 cells were stained with MitoSOX Red, which is a mitochondrial superoxide indicator, and the fluorescence intensity of MitoSOX was quantified. The amount of superoxide generated in mitochondria increased during apoptosis (treatment with H_2_O_2_) and ferroptosis (treatment with erastin) but decreased in response to SSSe NPs treatment. E) Quantification of MMP of H9C2 cells in each group, and the ratio was decreased during apoptosis (treatment with H_2_O_2_) and ferroptosis (treatment with erastin) but increased in response to the administration of SSSe NPs. F) Quantitative real‐time PCR analysis of mitochondrial (mt) DNA: mtDNA content was normalized to nuclear DNA. G) ATP concentrations were measured using an ATP assay kit. H–J) Ultrastructure of the myocardium: normal myocardial cell structure and mitochondrial structure in the sham group (H). Myocardial fibers were extensively damaged and mitochondrial structure was disordered in the saline‐treated group (I), while myocardial fibers and mitochondrial structure were intact in the SSSe NP‐treated group (J). All data are presented as the mean ± S.D. (*n* = 3). Statistical significance was calculated via one‐way ANOVA followed by a post hoc Bonferroni test. Ns *P* > 0.05, * *P* < 0.05, ** *P* < 0.01; *** *P* < 0.001.

Mitochondria are not only the energy source in cardiomyocytes but are also closely related to cardiomyocyte apoptosis and ferroptosis.^[^ ^27^, ^28^ ^]^ As mentioned previously, SSSe NPs passively targeted cardiomyocyte mitochondria in MI tissue (Figure 2J). The passive targeting of SSSe NPs to mitochondria was further demonstrated by incubating H9C2 cells with FITC‐SSSe NPs. As shown in **Figure** **4**A, FITC‐modified SSSe NPs efficiently entered cardiomyocytes under H_2_O_2_ stimulation and colocalized with red‐labeled mitochondria with a Pearson coefficient as high as 0.682 (Figure S23, Supporting Information). These experiments fully demonstrated that SSSe NPs achieved intrinsic mitochondrial targeting without resorting to complex or toxic modification of mitochondrial functional groups because of the dense mitochondrial distribution in cardiomyocytes. The passive mitochondrial targeting of SSSe NPs may be related to mitochondrial membrane depolarization under oxidative stress.^[^ ^29^ ^]^ Hypoxia‐induced mitochondria produced excess ROS, leading to the opening of the mitochondrial permeability transition pore and mitochondrial damage. More importantly, the mitochondrial membrane depolarization decreased its negative potential, which reduced the repulsive forces between SSSe NPs and mitochondria that contributes to the increased intrinsic mitochondrial targeting. In healthy cardiomyocytes, the Pearson coefficient of SSSe NPs and mitochondria was significantly less than its value in H_2_O_2_ treatment‐cardiomyocytes as seen in Figure S23, Supporting Information.

The protective effect of SSSe NPs on mitochondria was further investigated. Hypoxia increased the concentrations of H_2_O_2_ and O_2_.^−^ in mitochondria by interfering with the mitochondrial respiratory chain. Excessive mitochondrial ROS levels caused mitochondrial membrane depolarization and mitochondrial dysfunction (Figure 4B,E). SSSe NPs significantly scavenged excess mitochondrial ROS (Figure 4C,D) and excess ROS in the entire cell (Figure S24, Supporting Information) induced by H_2_O_2_ and erastin (a ferroptosis inducer). Moreover, SSSe NPs further ameliorated the depolarization of MMP induced by H_2_O_2_ and erastin (Figure 4B,E). mtDNA copy number is a key marker of mitochondrial integrity and was increased significantly in H9C2 cells after SSSe NPs treatment. Mitochondria in H9C2 cells treated with SSSe NPs maintained good integrity, as shown by TEM (Figure S25, Supporting Information). Mitochondria provide energy to cardiomyocytes by producing ATP. Therefore, the level of ATP production directly indicates mitochondrial function. SSSe NPs effectively ameliorated impaired mitochondrial ATP production induced by H_2_O_2_ and erastin (Figure 4G). Mitochondrial morphology was also further investigated in MI tissue. Compared with those in the sham groups (Figure 4H), mitochondria in untreated MI tissue had swollen, ruptured, and lost the cristae in the inner mitochondrial membrane (Figure 4I), indicating that the mitochondria had been destroyed (yellow arrows indicated damaged mitochondria, purple arrows indicated disrupted myofibers). As shown in Figure 4J, SSSe NPs effectively maintained the shape of myocardial mitochondria. These experiments and the results of the bioinformatics analysis showed that SSSe NPs passively targeted mitochondria in cardiomyocytes, effectively protected mitochondria in cardiomyocytes, and significantly maintained mitochondrial function.

### 3.5 SSSe NPs Reduce Cardiomyocyte Apoptosis and Ferroptosis

**Figure 5:**
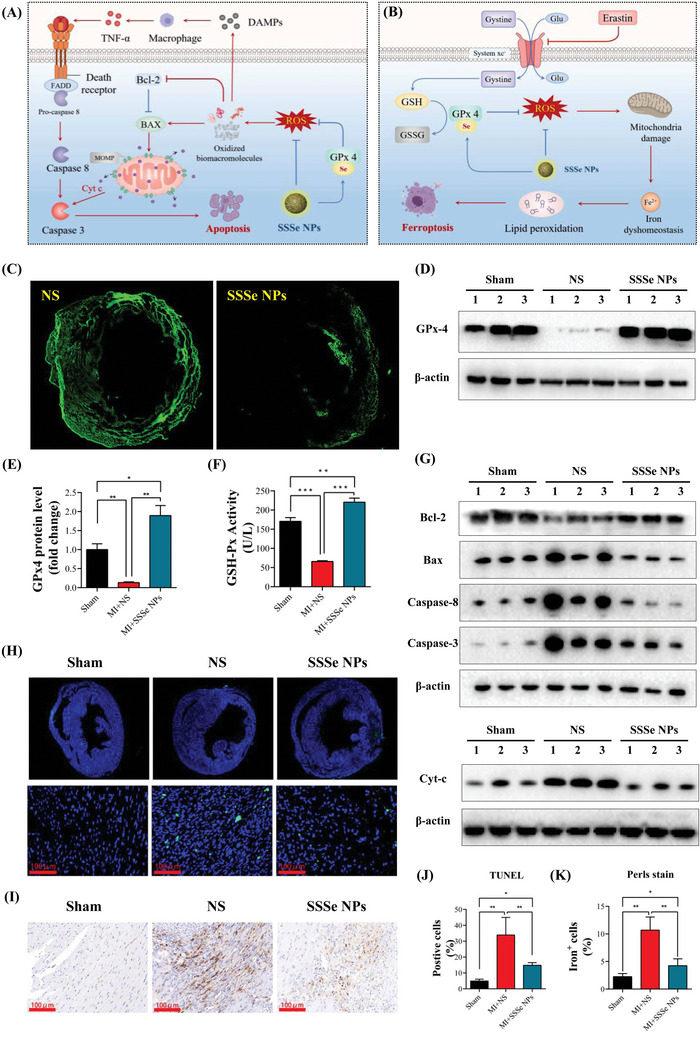
SSSe NPs administration alleviates apoptosis and ferroptosis in the myocardial cells of MI mice. A‐B) The proposed pathway by which SSSe NPs reduces apoptosis and ferroptosis in myocardial cells through GPx4‐related ROS scavenging. C) Measurement of ROS levels in heart tissues 7 days after MI using a DCFH‐DA probe (green). D‐E) Western blot showing GPx4 protein levels in each group 3 days after MI, and the relative protein levels of GPx4 were normalized to *β*‐actin. MI resulted in a significant decrease in GPx4 protein expression, and this effect was reversed by SSSe NPs. F) Tissue glutathione peroxidase (GSH‐Px) activity was assessed using a GSH‐Px assay kit. (*n* = 6). (G) Western blot analysis of apoptosis‐related proteins (Bcl‐2, Bax, caspase‐8, caspase‐3, and Cyt‐c) in heart tissues on day 3 after MI. All data are presented as the mean ± S.D. (*n* = 3). H) Representative immunofluorescent images revealed TUNEL‐positive cells in heart tissues on day 3 after MI. The images in the middle panel are magnified in the same areas as in the upper panel (green: TUNEL, blue: DAPI). (I) Immunocytochemical analysis of redox‐active iron deposits in heart tissues, as measured by a modified Perl's stain. J) Quantitation of TUNEL‐positive cells in heart tissues. All data are presented as the mean ± S.D. (*n* = 6). (K) Quantification of iron‐positive cells in heart tissues. All data are presented as the mean ± S.D. (*n* = 6). Statistical significance was calculated via one‐way ANOVA followed by a post hoc Bonferroni test. Ns *P* > 0.05, * *P* < 0.05, **P < 0.01; *** *P* < 0.001.

Excessive ROS in MI tissue could damage many biomolecules, such as proteins, lipids, and nucleic acids, and further damage the mitochondrial respiratory chain to release Cyt C and induce caspase 3‐based to cause cardiomyocyte apoptosis.^[^ ^30^, ^31^ ^]^ DAMPs from injured cardiomyocytes stimulated inflammatory cells to release various inflammatory factors, such as TNF‐*α*, and induced cardiomyocyte apoptosis via the caspase 8 pathway^[^ ^32^ ^]^ (**Figure** **5**A). In addition, ROS caused an imbalance in iron metabolism to induce cardiomyocyte ferroptosis by damaging mitochondria^[^ ^33^ ^]^ (Figure 5B). Given the important role of ROS in cardiomyocyte apoptosis and ferroptosis, the cardioprotective effect of SSSe NP‐mediated ROS reduction as investigated. SSSe NP‐treated MI tissues maintained lower ROS levels than untreated tissue, and the ROS levels in the SSSe NP‐treated groups remained low on day 7 (Figure 5C), suggesting that the sustained elimination of ROS may be due to the high level of GP_X_4 converted from SSSe NPs in MI tissue. GPx4 expression was further investigated in MI tissue. As shown in Figure 5D, the expression level of GPx4 in MI tissues was less than that in the sham group because the ROS burst at the early stage of MI damaged the GPx4 antioxidant system, resulting in persistently low expression of GPx4 during the middle and late stages of MI. SSSe NPs greatly promoted the expression of GPx4 even more than sham treatment (Figure 5D,E). Moreover, the activity of GPx4 in the SSSe NP‐treated group was much higher than that in the untreated and sham groups (Figure 5F). Combined with the Se levels in MI tissue (Figure S11, Supporting Information), these results fully demonstrate that the Se in SSSe NPs is been converted to GPx4 at the MI site.

SSSe NPs were further shown to reduce cardiomyocyte apoptosis through the caspase‐3 and caspase‐8 pathways, as indicated by the expression levels of key proteins in these pathways in MI tissues, including Bax, Bcl‐2, cyt‐c, caspase‐3, and caspase‐8. The expression levels of Bax, cyt‐c, caspase‐3 and caspase‐8 in the NS group were much higher than those in the sham group, and the antiapoptotic factor Bcl‐2 was significantly decreased. As expected, SSSe NPs greatly inhibited cardiomyocyte apoptosis by interfering with the caspase‐3 and caspase‐8 pathways (Figure 5G, Figure S25, Supporting Information). Moreover, terminal deoxynucleotidyl transferase dUTP nick‐end labeling (TUNEL) was used to identify and quantify apoptotic cells in MI tissue. As shown in Figure 5H,J, SSSe NPs significantly reduced the number of apoptotic cells in MI tissue. Ferroptosis, another important form of cardiomyocyte death in MI, was closely associated with decreased GPx4 expression and iron metabolism imbalance. SSSe NPs effectively reduced Fe deposition to restore iron homeostasis in MI tissue, as shown by Prussian blue staining (Figure 5I, Figure K, Figure S28, Supporting Information). The mechanism of Fe homeostasis recovery by SSSe NPs was further investigated. Transferrin receptor and ferroportin are key proteins that affect iron homeostasis in cardiomyocytes. Transferrin receptor transported extracellular Fe^3+^ into the cell to form Fe^2+^, and ferroportin was responsible for transporting intracellular Fe^2+^ out of the cell.^[^ ^34^ ^]^ Iron homeostasis was maintained through the coordination of these factors in cardiomyocytes (Figure S27, Supporting Information). The expression of the transferrin receptor was significantly increased after MI, which promoted the entry of Fe^3+^ into cardiomyocytes to induce ferroptosis (Figure S29A,C, Supporting Information). After treatment with SSSe NPs, the expression of ferroportin was increased compared with that in the NS group (Figure S29B,D, Supporting Information). As a result, SSSe NPs promoted excessive Fe^2+^ efflux from cardiomyocytes and significantly reduced ferroptosis in MI tissue. Another marker of ferroptosis was malondialdehyde (MDA) due to ROS‐induced lipid peroxidation. GPx4 effectively reduced lipid peroxidation to significantly inhibit ferroptosis. As shown in Figure S29E, Supporting Information, was GPx4 converted from SSSe NPs and significantly reduced MDA levels in MI tissue. These results proved that SSSe NPs significantly reduced apoptosis and ferroptosis in cardiomyocytes in MI site through a self‐sustaining antioxidant effect.

### 3.6 SSSe NPs Reduce Inflammation and Fibrosis

**Figure 6:**
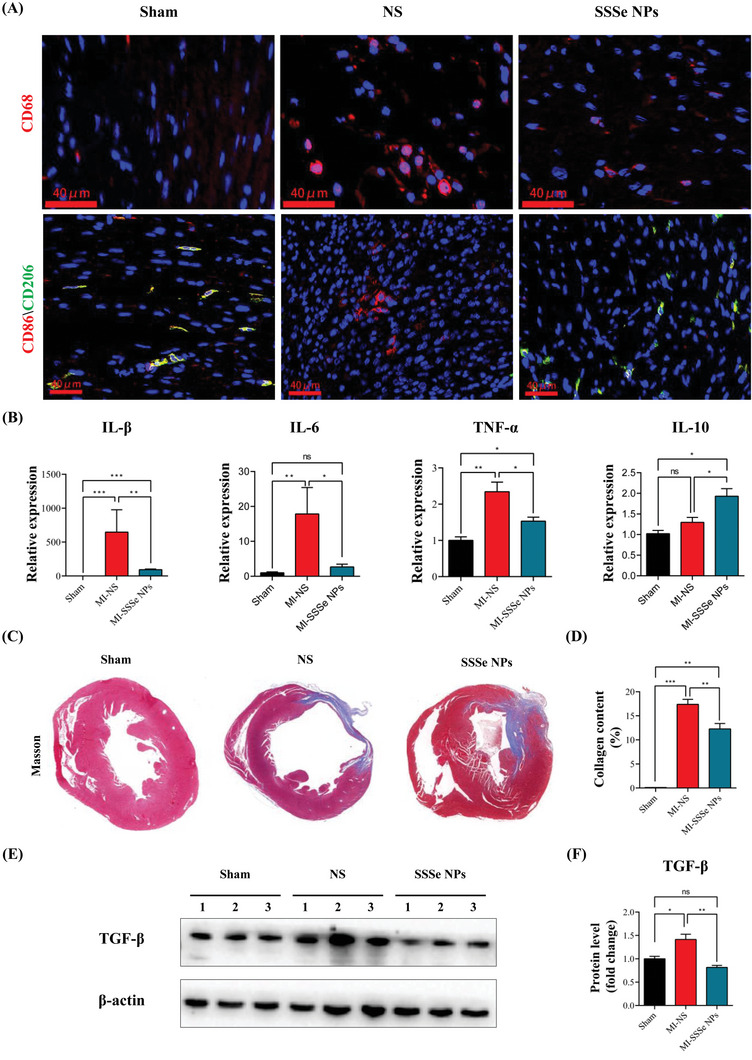
SSSe NPs administration alleviates myocardial inflammation and fibrosis in MI mice. A) Immunofluorescence staining of CD68 (macrophage marker, red) with the mannose receptor (CD206, M2 marker, green) and (CD86, M1 marker, red) in heart tissues. The nuclei were stained with DAPI (blue). (B) RT‒qPCR analysis of the mRNA levels of the proinflammatory cytokines IL‐1*β*, IL‐6, and TNF‐*α*, as well as the anti‐inflammatory cytokine IL‐10. (C) Representative images of Masson trichrome staining of infarcted cardiac tissue showing fibrosis on day 14 after MI. D) Quantification of the area of fibrosis in the heart in each treatment group. All data are presented as the mean ± S.D. (*n* = 6). E–F) Western blot showing the expression of TGF‐*β* on day 3 after MI. The protein level was significantly decreased after treatment with SSSe NPs compared to saline. All data are presented as the mean ± S.D. (*n* = 3). Statistical significance was calculated via one‐way ANOVA followed by a post hoc Bonferroni test. Ns *P* > 0.05, * *P* < 0.05, **P < 0.01; *** *P* < 0.001.

Excessive ROS induced inflammatory cell infiltration through the production of DAMPs and formed a vicious cycle of inflammation and ROS production in MI. Inflammation and fibrosis in MI tissue were closely related to macrophage infiltration and polarization.^[^ ^35^ ^]^ As shown in **Figure** **6**A, CD86‐positive proinflammatory M1 cells (Red) were significantly increased and CD206‐positive anti‐inflammatory M2 macrophages (Green) were significantly decreased in the NS group compared with the sham group. SSSe NPs effectively reduced macrophage infiltration (CD68) and increased the M2/M1 ratio in MI tissue (Figure 6A, Figure S30, Supporting Information). Moreover, the expression of inflammatory factors such as IL‐1*β*, IL‐6, and TNF‐*α* in MI tissue was significantly reduced in the nanoparticle‐treated group, while the expression of anti‐inflammatory factors such as IL‐10 was significantly increased (Figure 6B). SSSe NP‐induced depolarization of macrophages was further investigated at the cellular level in RAW264.7 cells. As shown in Figure S31, Supporting Information, RAW264.7 cells were polarized to the M1 type by lipopolysaccharides (LPS), while SSSe NPs effectively depolarized RAW264.7 cells.

Fibrosis is the main cause of poor myocardial remodeling in the middle and late stages of MI, and pathological collagen deposition replaced myocardial cells, leading to abnormal cardiac function.^[^ ^36^ ^]^ As shown in Figure 6C,D, the collagen level in the SSSe NP‐treated group was much less than that in the NS group (17.37 ± 1.046 vs 12.17 ± 1.131, %), as shown by Masson staining, indicating that SSSe NPs effectively reduced fibrosis in MI tissue. Cardiac fibrosis was associated with the secretion of TGF‐*β* by inflammatory cells. MI tissues had low TGF‐*β* expression levels after SSSe NPs treatment (Figure 6E,F). Overall, SSSe NPs significantly reduced inflammation and fibrosis in MI tissue by inhibiting inflammatory cell infiltration and polarization, which was also consistent with the sequencing results (Figure 3I).

### 3.7 SSSe NPs Promote Angiogenesis

**Figure 7:**
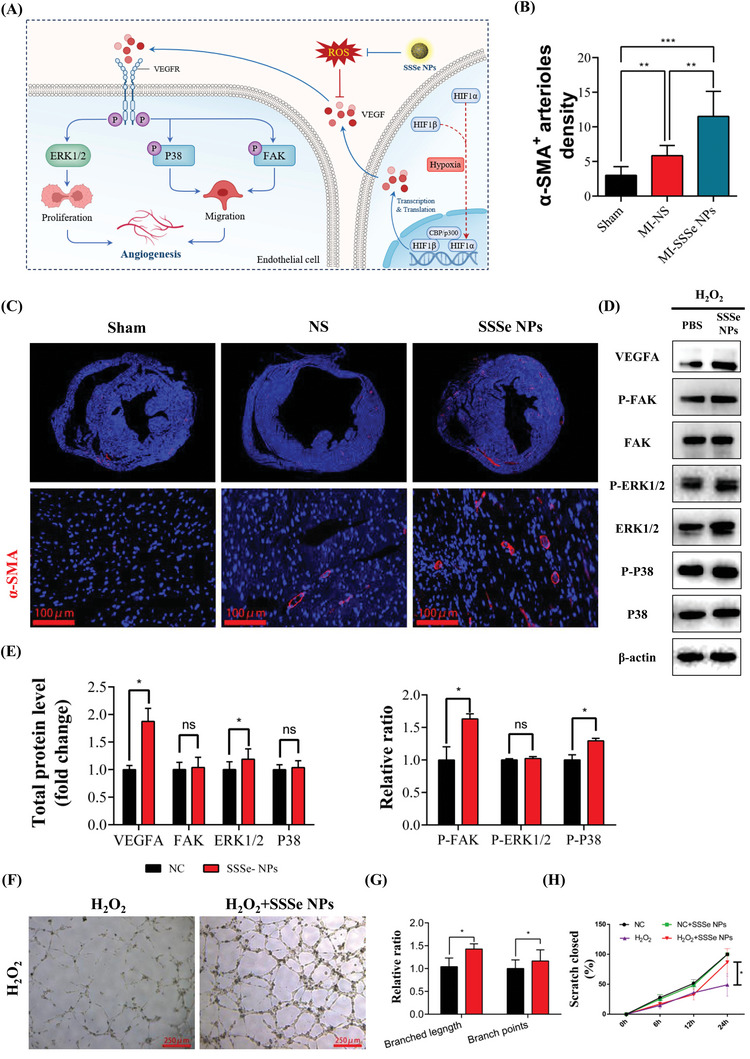
SSSe NPs administration improves angiogenesis in MI hearts A) The proposed pathway by which SSSe NPs activate angiogenesis in endothelial cells by regulating ROS production. B) Quantification of the numbers of *α*‐SMA‐positive arterioles. All data are presented as the mean ± S.D. (*n* = 6) C) Representative immunofluorescent staining of *α*‐SMA (red) in the cardiac infarct junction region (bottom) on day 14 after MI, and the nuclei were stained with DAPI (blue). D–E) Representative Western blot showing VEGF A, p‐FAK, FAK, p‐ERK1/2, ERK1/2, p‐P38, and P38 expression in HUVECs treated with PBS or SSSe NPs under H_2_O_2_ injury conditions. All data are presented as the mean ± S.D. (*n* = 3). F) Typical images of the tube formation assay. G) Angiogenesis was measured by an in vitro tube formation assay, and the bar graph shows the percentage of branch lengths and points. All data are presented as the mean ± S.D. (*n* = 3). H) Migration was measured by in vitro scratch experiments; the bar graph shows the percentage of scratch closure. All data are presented as the mean ± S.D. (*n* = 3). Statistical significance was calculated via one‐way ANOVA followed by a post hoc Bonferroni test. Ns *P* > 0.05, * *P* < 0.05, **P < 0.01; *** *P* < 0.001.

Angiogenesis is one of the keys to MI tissue repair because new blood vessels can effectively restore the O_2_ and nutrient supply for MI tissue and restore cardiac function.^[^ ^37^ ^]^ The hypoxic microenvironment induced the expression and stability of HIF‐*α* in MI tissue, which promoted the transcription and secretion of VEGF A in MI tissue. Subsequently, VEGF A promoted endothelial cell proliferation and migration after binding to VEGF receptor 2 (VEGFR 2) on vascular endothelial cells.^[^ ^38^ ^]^ However, continuous ROS production after MI impaired the structure and function of endothelial cells. Neovascularization in MI was somewhat enhanced compared to that in normal tissue but was not sufficient to rescue the necrotic myocardium or significantly improve myocardial function (**Figure** **7**A). The effect of SSSe NPs on angiogenesis was investigated by *α*‐smooth muscle actin (*α*‐SMA, a vascular smooth muscle‐specific biomarker) immunostaining. As shown in Figure 7B,C, SSSe NPs significantly increased new blood vessels, and SSSe NPs increased the density of *α*‐SMA+ vessels by ≈two‐fold compared to that in untreated MI tissue.

The angiogenic mechanism of SSSe NPs was further investigated through some key angiogenesis proteins, such as VEGF A, extracellular signal‐regulated kinase 1/2 (ERK1/2), focal adhesion kinase (FAK) and p38 (Figure 7A). VEGF A activated ERK1/2 to promote endothelial cell proliferation and activated P38 and FAK to promote endothelial cell migration. Under high concentrations of H_2_O_2_ (simulating the MI tissue environment), the secretion of VEGF A by injured endothelial cells was significantly reduced. In contrast, SSSe NPs promoted the expression of VEGF A, further promoted the expression of ERK1/2, which is involved in proliferation; SSSe NPs also activated FAK and P38, which are involved in endothelial cell migration (Figure 7D,E, Figure S32D, Supporting Information). The effect of SSSe NPs on endothelial cell function was also investigated. As shown in Figure 7F,G, SSSe NPs promoted endothelial cell tube formation and endothelial cell migration in response to H_2_O_2_ (Figure 7H, Figure S32, Supporting Information). In addition, SSSe NPs effectively rescued H_2_O_2_‐induced endothelial cell damage (Figure S33, Supporting Information).

### 3.8 Discussion and Conclusion

In normal cardiomyocytes, GPx4 is key part of an endogenous antioxidant system that sustainably eliminates ROS and maintains ROS homeostasis.^[^ ^39^ ^]^ In this study, a sustainable antioxidant strategy was proposed for the first time to treat MI with SSSe NPs targeting the differential properties of three distinct pathological progression stages of ROS. SSSe NPs use C–Se bonds to embed Se elements into the carbon skeleton, which can efficiently scavenge a variety of ROS, very efficiently scavenge ROS bursts during the early stage of MI, and be converted into GPx4 for continuous on‐demand scavenging during inflammation and fibrosis. SSSe NPs protected mitochondria to reduce myocardial apoptosis and ferroptosis, inhibited inflammation and fibrosis, and ultimately promoted angiogenesis in MI tissue. Although this promising therapeutic effect is in the preclinical stage, SSSe NPs, which are a highly promising treatment for MI, are expected to be clinically translated in the future. Many nanomedicines have shown excellent efficacy in preclinical studies of MI but are difficult to translate into clinical use. In general, this barrier is mainly caused by the following two issues.

First, the potential toxicity of nanomedicines is important. For example, although ceria nanomedicine has been widely used as an antioxidant therapy, its potential toxicity greatly limits its clinical application.^[^ ^40^ ^]^ The toxicity of nanomedicine is mainly considered from three aspects: biocompatibility, biodegradability, and excretion. SSSe NPs were prepared from a selenium‐containing amino acid and did not contain any toxic elements (Figure S1, Supporting Information). SSSe NPs are physiologically stable because Se is intercalated into the carbon skeleton through C–Se covalent bonds. High doses of SSSe NPs did not have any effect on the liver, kidney function, or blood system (Figures S34–S35, Supporting Information) or affect vital organs (Figure S36, Supporting Information). In addition, SSSe NPs were gradually degraded in vivo (Figure 2G) and excreted through the kidneys (Figures S8,S9, Supporting Information). These data demonstrated the excellent biocompatibility, biodegradability, and excretion of SSSe NPs.

Second, the complex components of nanomedicines should be considered. For example, to achieve good efficacy, nanomedicines have been increasingly designed to be very complex to target specific tissues and organelles.^[^ ^41^ ^]^ Different from the laboratory setting, nanomedicine requires standardized management of components and preparation methods for large‐scale manufacturing.^[^ ^42^ ^]^ However, the synergy of components and the complexity of the preparation make standardization exponentially more difficult as the number of components in nanomedicines increases. SSSe NPs specifically accumulated in MI tissue and entered cardiomyocyte mitochondria without the addition of any targeting groups. Therefore, SSSe NPs could be easily produced and standardized on a large‐scale in the next clinical translation process.

In conclusion, SSSe NPs had a surprisingly excellent therapeutic effect on MI due to their self‐sustaining antioxidant properties. In addition, SSSe NPs have great clinical translation value in the treatment of MI and provide a distinctive inspiration and reference for the treatment of other diseases, such as cerebral infarction, which has similar pathological characteristics.

## Conflict of Interest

The authors declare no conflict of interest.

## Supporting information

Supporting Information

## Data Availability

The data that support the findings of this study are available from the corresponding author upon reasonable request.
