# Supplementary material for: A Self‐Sustaining Antioxidant Strategy for Effective Treatment of Myocardial Infarction
Source: Adv Sci (Weinh). 2022 Dec 25;10(5):2204999. doi: 10.1002/advs.202204999 (PMC9929116; doi:10.1002/advs.202204999)
Supplement: Supplementary file 1 — Supporting Information [file ADVS-10-2204999-s001.pdf]

ADVANCED SCIENCE
Open Access

Supporting Information

for *Adv. Sci.*, DOI 10.1002/advs.202204999

A Self-Sustaining Antioxidant Strategy for Effective Treatment of Myocardial Infarction

Quan Sun, Hongqin Ma, Jiaxiong Zhang, Baiyang You, Xiaohui Gong, Xiaolin Zhou, Jin Chen, Guogang Zhang, Jia Huang, Qiong Huang, Yurong Yang, Kelong Ai and Yongping Bai**

Supporting Information

A Self-Sustaining Antioxidant Strategy for Efficient Treatment of Myocardial Infarction

Quan Sun[1,2], Hongqin Ma[1,2], Jiaxiong Zhang[1,2], Baiyang You[5], Xiaohui Gong[2,6], Xiaolin Zhou[1,2], Jin Chen[1], Guogang Zhang[1,2,6], Jia Huang[3,4,], Qiong Huang[2,7], Yurong Yang[2,7], Kelong Ai[3,4,*], Yongping Bai[1,2,*].

1. Department of Geriatric Medicine, Coronary Circulation Center, Xiangya Hospital, Central South University, Changsha, Hunan, P.R. China.
2. National Clinical Research Center for Geriatric Disorders, Xiangya Hospital, Central South University, Changsha, Hunan, P.R.China.
3. Xiangya School of Pharmaceutical Sciences, Central South University, Changsha, Hunan, P.R. China.
4. Hunan Provincial Key Laboratory of Cardiovascular Research, Xiangya School of Pharmaceutical Sciences, Central South University, Changsha, Hunan, P.R. China.
5. Cardiac Rehabilitation Center, Department of Rehabilitation, Xiangya Hospital of Central South University, Changsha, Hunan, P.R. China.
6. Department of Cardiology, The Third Xiangya Hospital, Central South University, Changsha, Hunan, P.R. China.
7. Department of Pharmacy, Xiangya Hospital, Central South University, Changsha, 410008, China

* Corresponding authors:
Dr. Yongping Bai, e-mail: baiyongping@csu.edu.cn
Dr. Kelong Ai, e-mail: aikelong@csu.edu.cn

Materials

Selenocystine, NaOH, dopamine hydrochloride, tris(hydroxymethyl)aminomethane and FITC were purchased from Macklin Company (Shanghai). CCK-8 and SOD assay kit were purchased from Dojindo Molecular Technologies (Kumamoto, Japan). TNF-α and IL-6 ELISA kits were purchased from Elabscience Biotechnology (Houston, Texas, USA). A TUNEL assay kit (C10617), Caspase 3 polyclonal antibody (PA577887), Goat anti-Rabbit IgG (H+L) Highly Cross-Adsorbed secondary antibody Alexa Fluor 488 (A11034), Goat anti-Rabbit IgG (H+L) Cross-Adsorbed secondary antibody Alexa Fluor 555 (A21428), MitoSOX™ Red Mitochondrial Superoxide Indicator (M36008) and ProLong™ Glass Antifade Mountant with NucBlue™ Stain (P36983) were obtained from Thermo Fisher Scientific (Carlsbad, CA, USA). Enhanced mitochondrial membrane potential assay kit with JC-1(C2003S), Hoechst 33342 Staining Solution for Live Cells (C1028), and MDA were purchased from Beyotime Biotechnology (Shanghai, China). COX-2 antibody (CST,12282S) and F4/80 antibody (70076S) were purchased from Cell Signaling Technology (Danvers, MA, USA). BAX (ab32503). Bcl-2 antibody (BF9103) were obtained from Affinity Bioscience (Jiangsu, China). Cyt c antibody (d10933-1-AP) were purchased from Proteintech (Rosemont, IL, USA).

Characterization

TEM images were taken by using a TECNAI G2 high-resolution transmission electron microscope. XPS measurements were conducted with a VG ESCALAB MKII spectrometer. The XPSPEAK software (Version 4.1) was used to deconvolute the narrow-scan XPS spectra of the W 4f of the samples, using adventitious carbon to calibrate the C1s binding energy (284.5 eV). FTIR was recorded on a Bruker Vertex 70 spectrometer (2 cm^{-1}). UV/Vis spectra were collected using a VARIAN CARY 50 UV/Vis spectrophotometer. The fluorescence spectra were determined by using an F98 spectrofluorometer. The ICP measurements were conducted using the Thermo/Jarrell Ash Advantage Atomscan Inductively Coupled Argon Plasma Spectrometer.

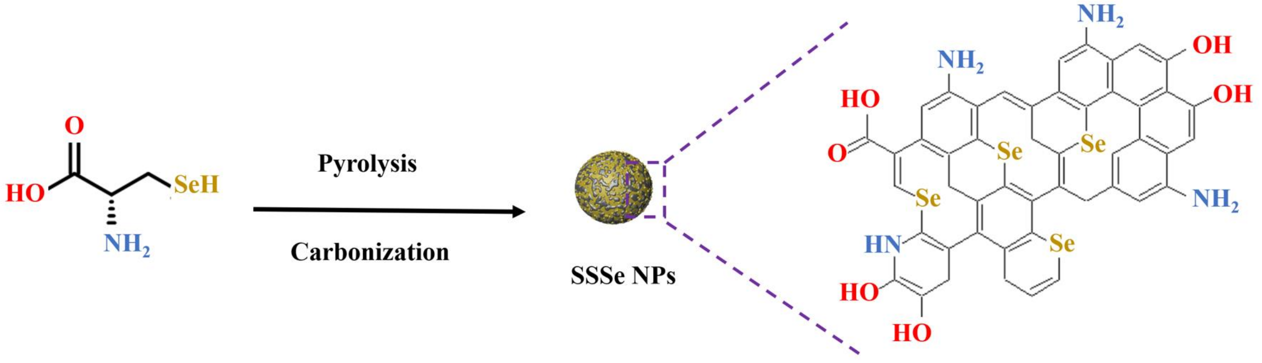

Figure S1 Schematic illustration of SSSe NP preparation.

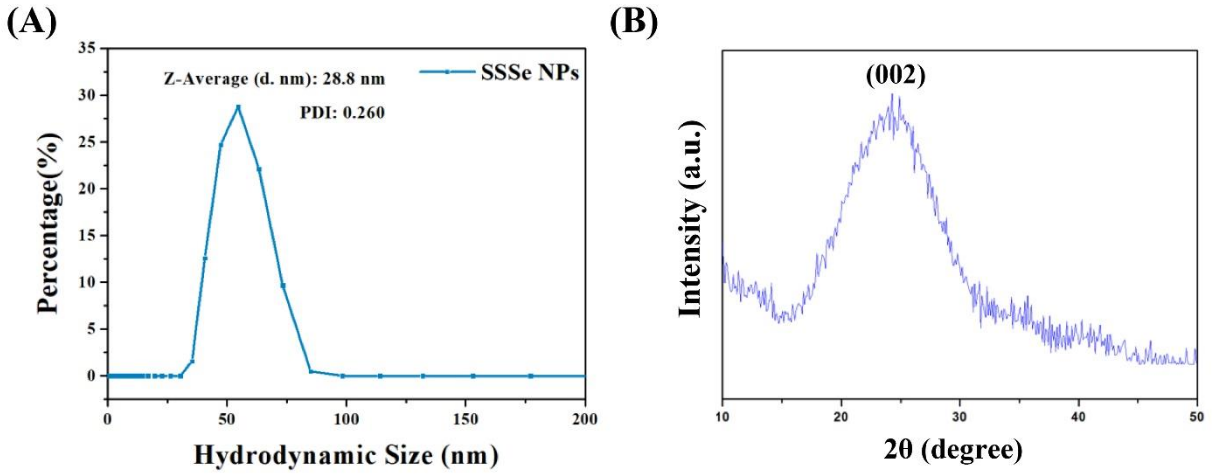

Figure S2 (A) X-ray diffraction (XRD) pattern of SSSe NPs. (B) Hydrodynamic diameter distribution of SSSe NPs.

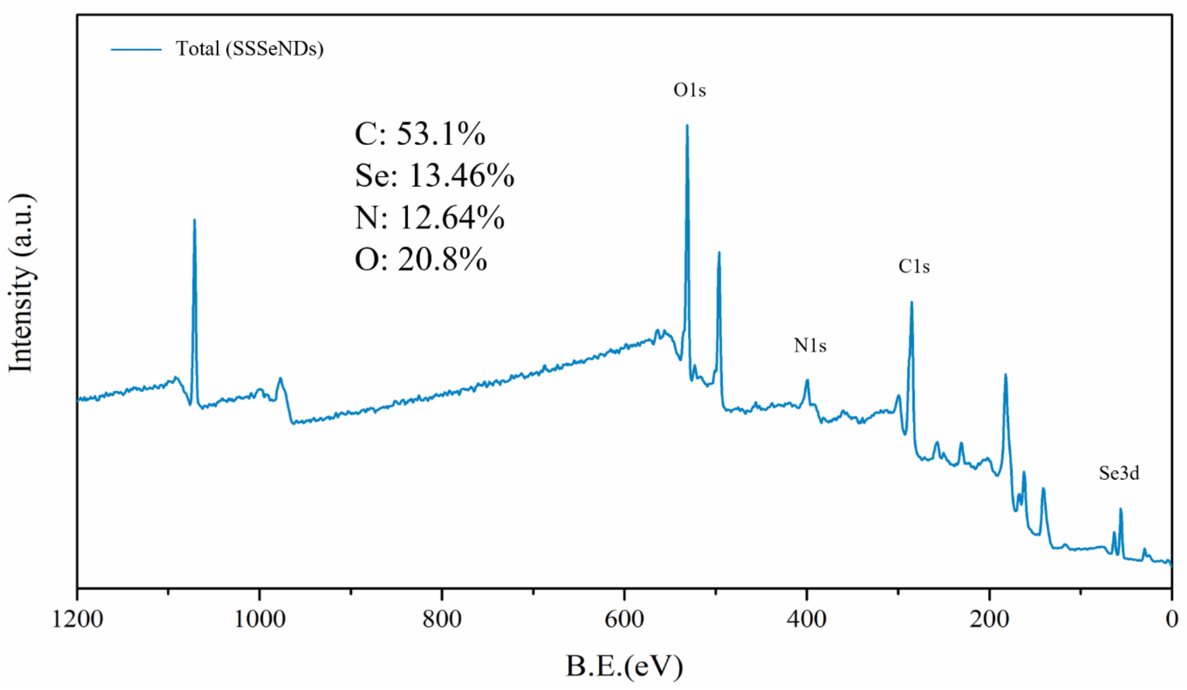

Figure S3 XPS spectrum of SSSe NPs.

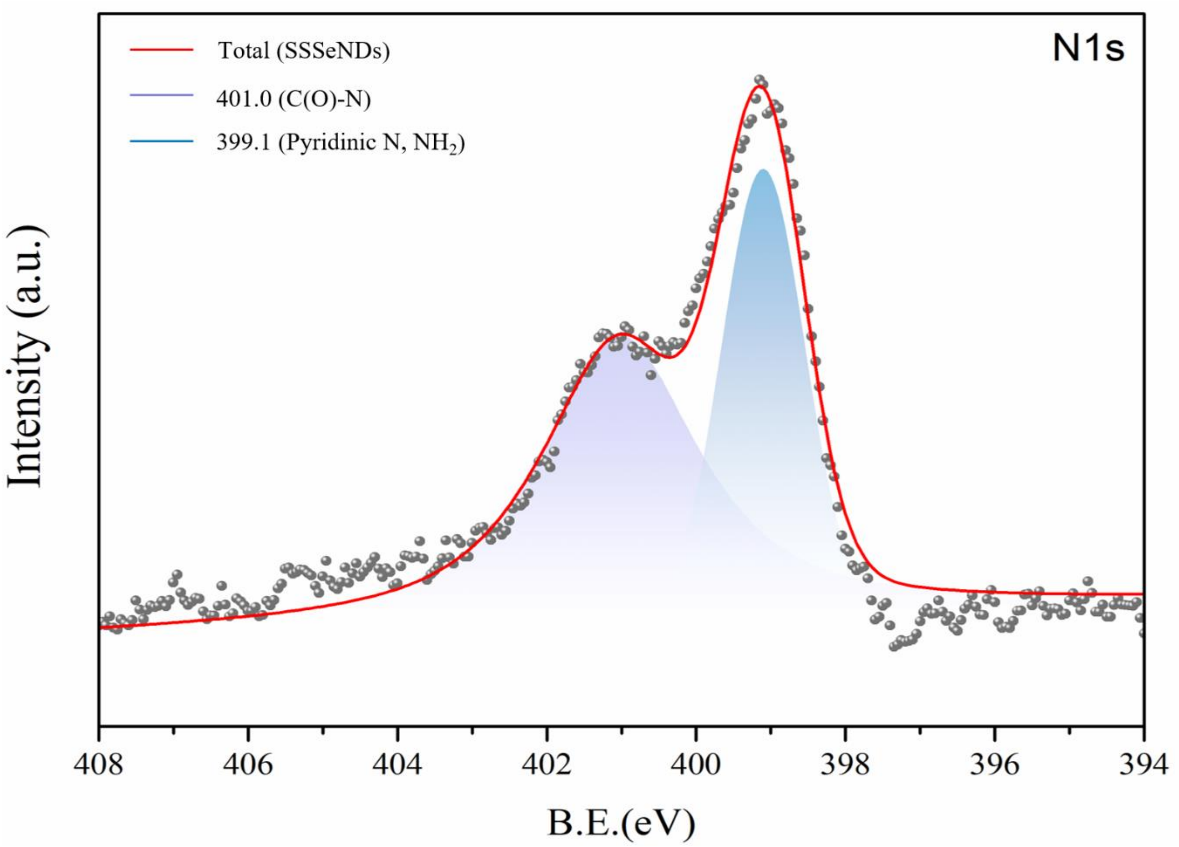

Figure S4 XPS spectrum of N1s in SSSe NPs.

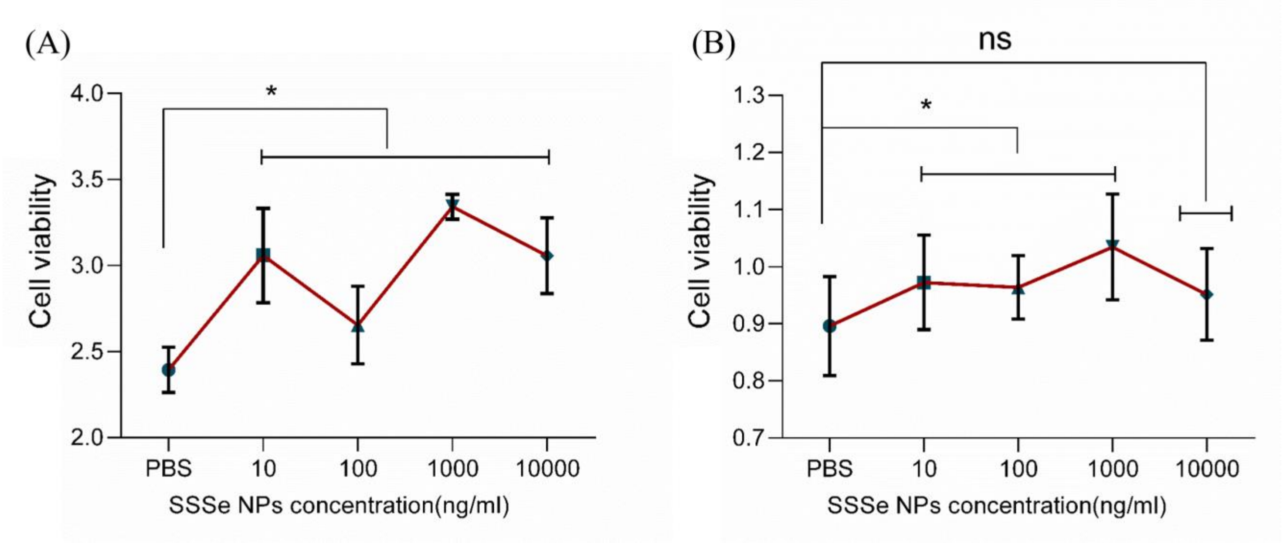

Figure S5 (A) Viability of H9C2 cells treated with different concentrations of SSSe NPs, as determined by a CCK-8 kit. (B) Viability of HUVECs treated with different concentrations of SSSe NPs, as determined by a CCK-8 kit. All data are presented as the mean ± S.D. (n = 6). (* *P*<0.05, Student's t test).

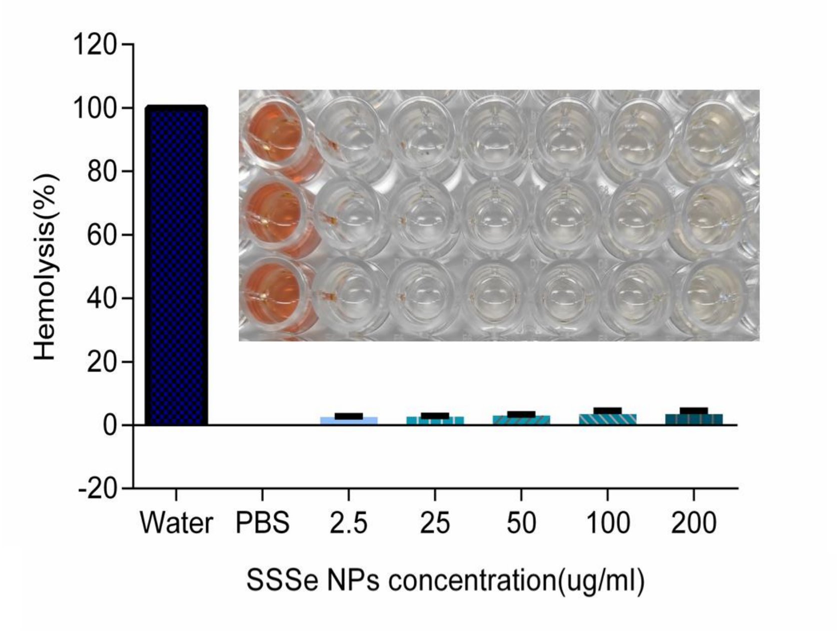

Figure S6 Hemolytic activity of different concentrations of SSSe NPs. The positive control, which showed 100% hemolysis, contained water and erythrocytes only. The negative control was a measure of spontaneous hemolysis and contained PBS and erythrocytes only. The bar graph shows the quantification of the relative hemolysis degree. All data are presented as the mean ± S.D. (n = 3).

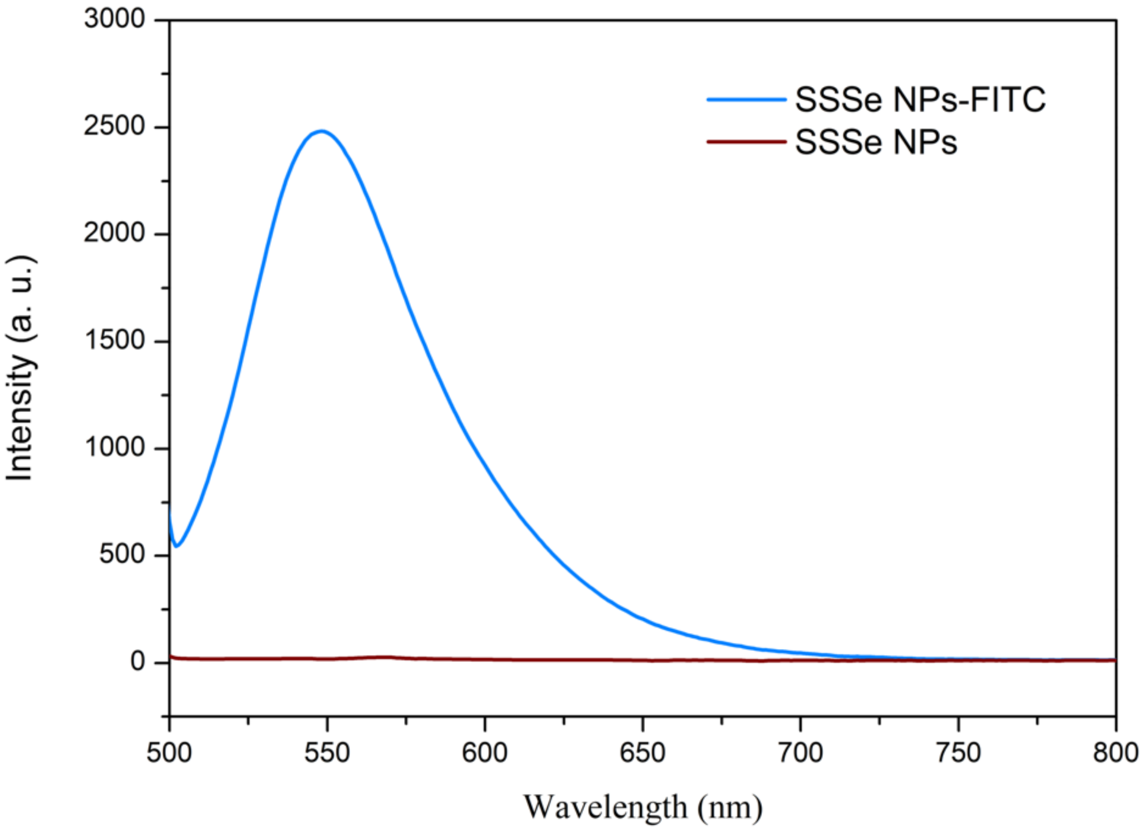

Figure S7 Fluorescence spectrum of SSSe NPs and SSSe NPs-FITC.

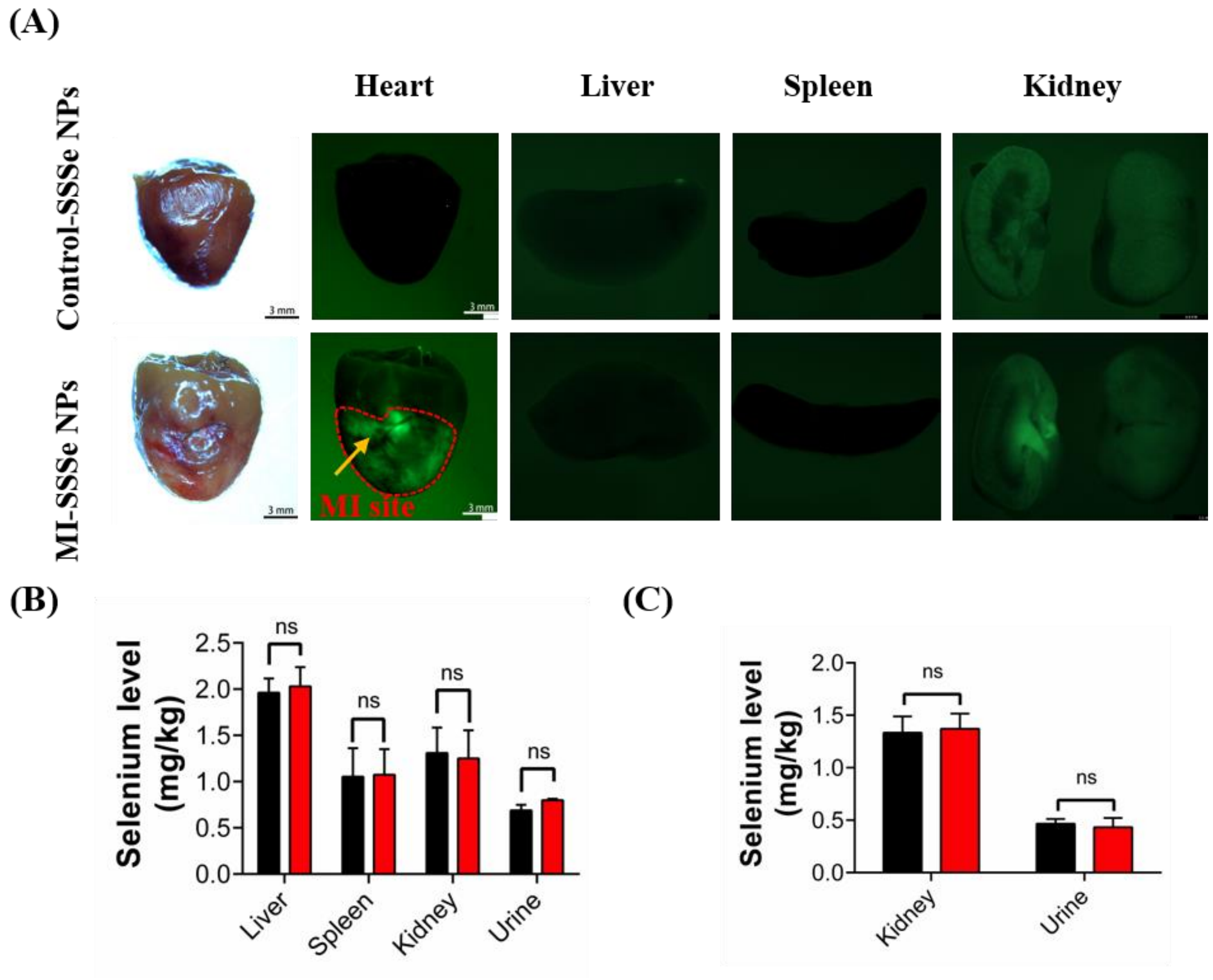

Figure S8 (A) Representative ex vivo fluorescence imaging of other mouse organs. (B) Quantification of the selenium content in organs and urine was determined by ICP at day 3. (C) Quantification of the selenium content in kidney and urine was determined by ICP at day 14. All data are presented as the mean ± S.D. (n = 3-5). Statistical significance was calculated by an unpaired two-tailed Student's t test. ns $P > 0.05$, * $P < 0.05$, **$P < 0.01$; *** $P < 0.001$.

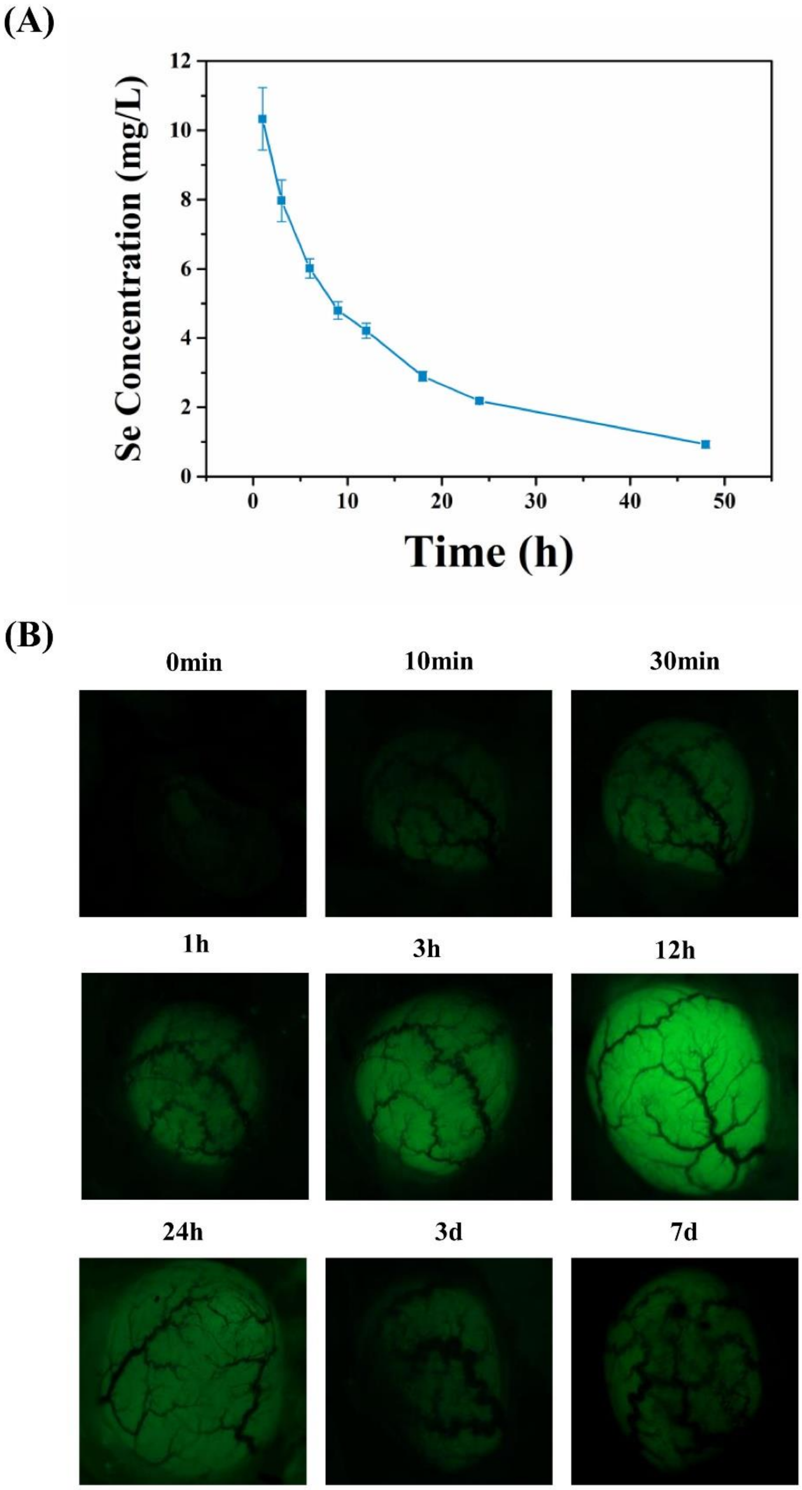

Figure S9 (A) The blood half-life of SSSe NPs. (B) Representative ex vivo fluorescence imaging of bladder at different intervals after drug administration.

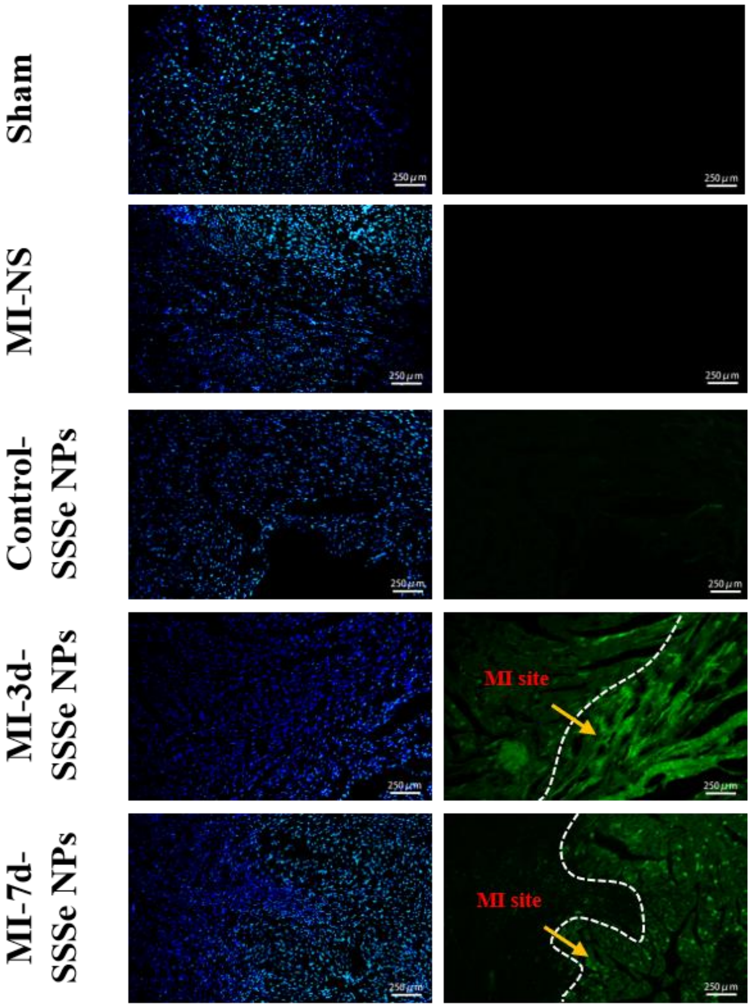

Figure S10 Representative fluorescence imaging of frozen sections of mouse hearts in the different groups showing FITC-labeled SSSe NPs (green) located at the infarct region, and the nuclei were stained with DAPI (blue). The control-SSSe NP group (without AMI surgery but receiving SSSe NP injection) did not show remarkable fluorescence.

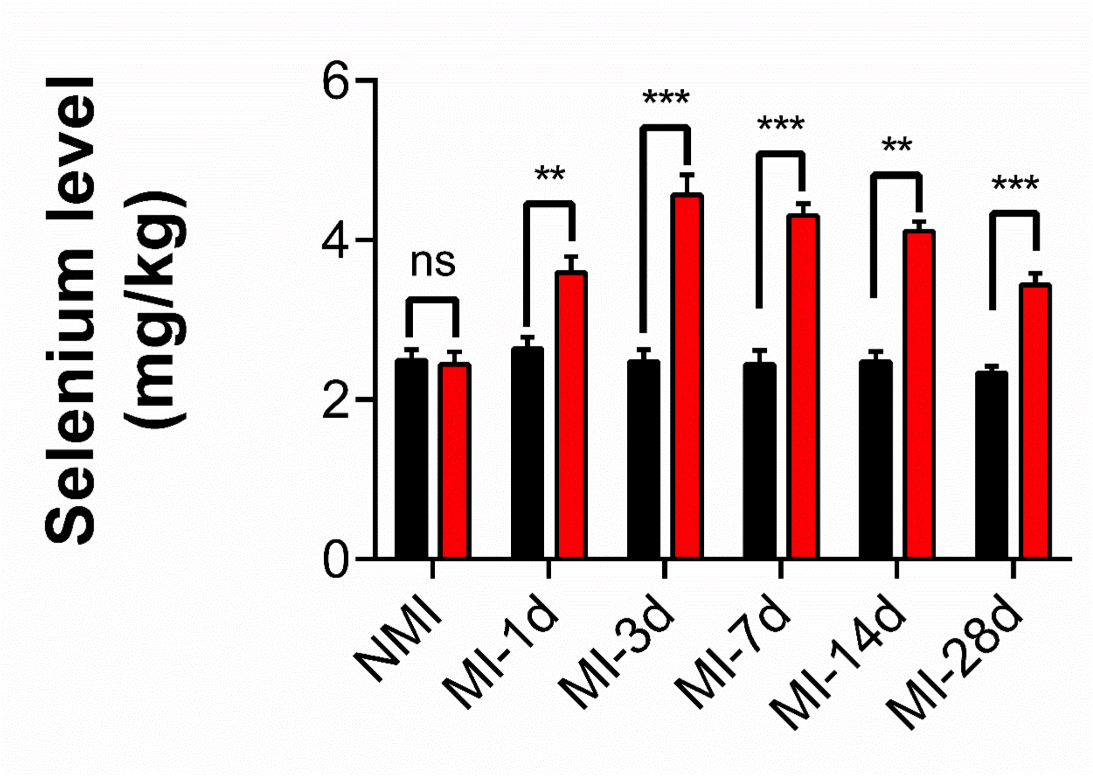

Figure S11 Quantification of the selenium content in heart tissues after the treatment was determined by ICP. All data are presented as the mean ± S.D. (n = 5). Statistical significance was calculated by an unpaired two-tailed Student's t test. ns $P > 0.05$, * $P < 0.05$, **$P < 0.01$; *** $P < 0.001$.

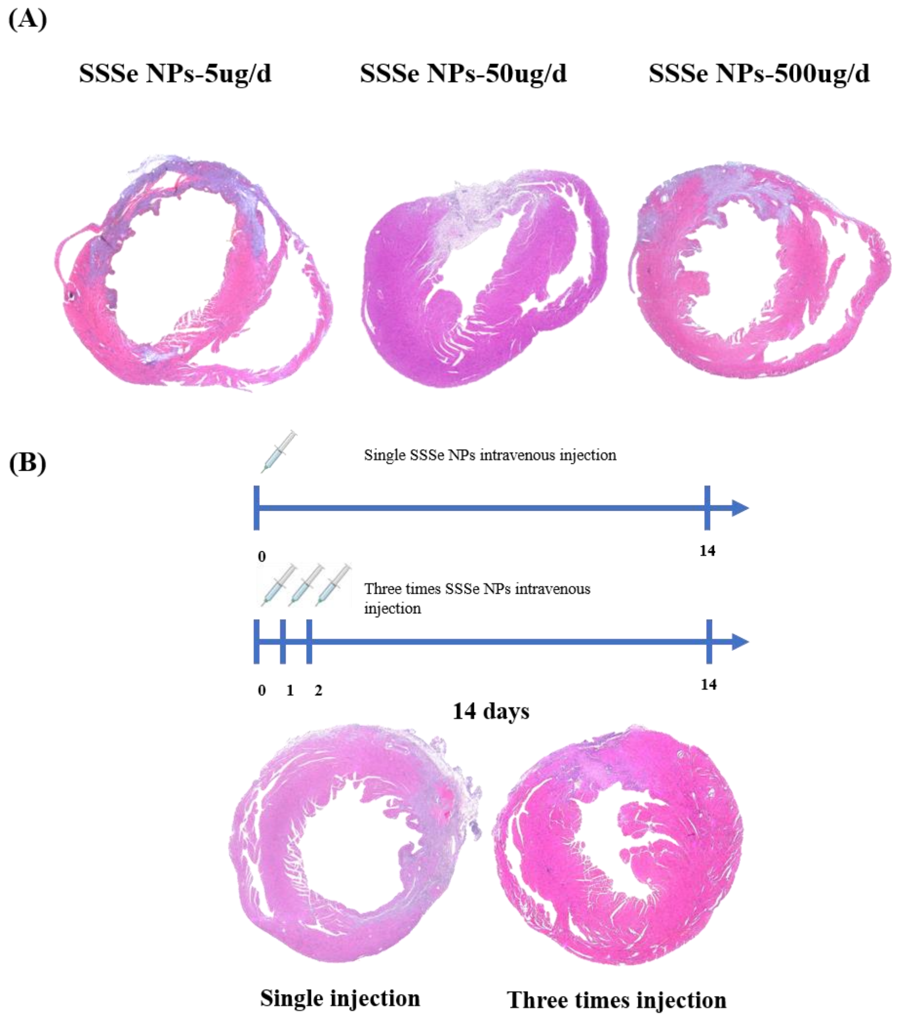

Figure S12 (A) Representative HE staining images from mice treated with different concentration of SSSe NPs. (B) Representative HE staining images after SSSe NP administration by a single injection or three injections after MI surgery.

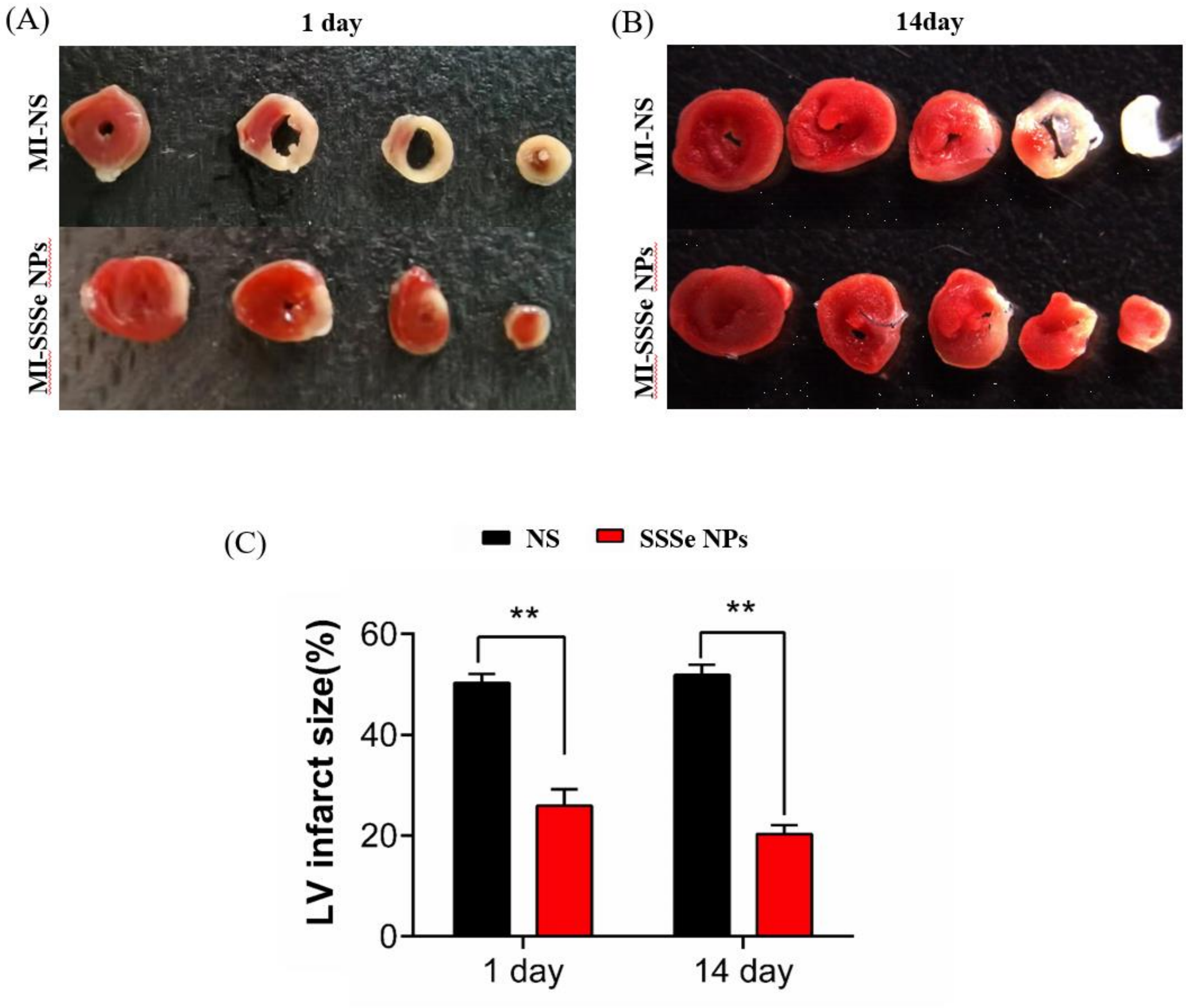

Figure S13 (A) TTC staining of infarcted cardiac tissues showing infarction size on day 1 and day 14 after MI. (C) Infarction sizes are expressed as a percentage of the whole heart area of MI-NS and MI-SSSe NP-treated mice. Statistical significance was calculated by an unpaired two-tailed Student's t test. ns $P > 0.05$, * $P < 0.05$, ** $P < 0.01$; *** $P < 0.001$.

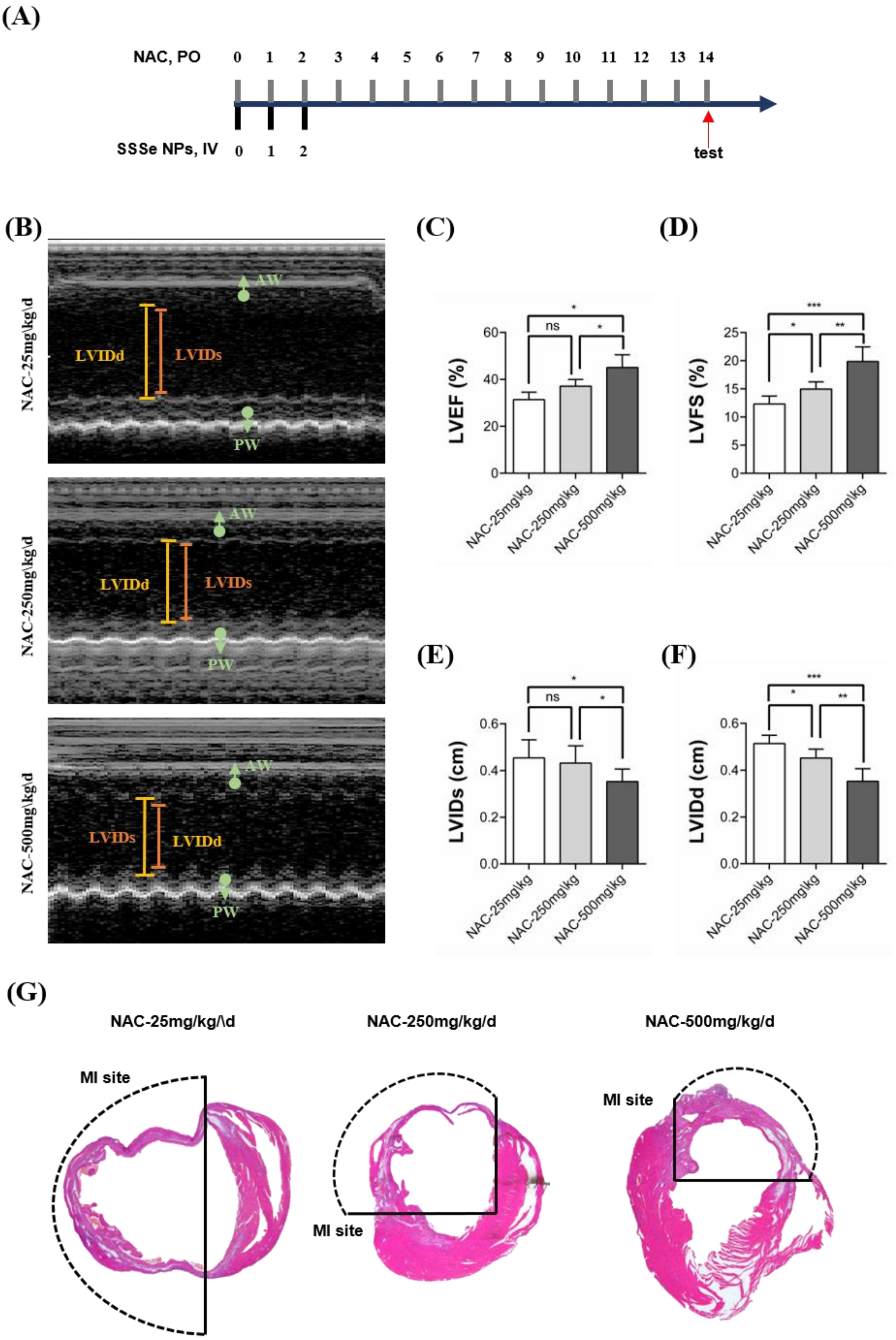
(A)
NAC, PO
0 1 2 3 4 5 6 7 8 9 10 11 12 13 14
SSSe NPs, IV
0 1 2
test
(B)
NAC-25mg\kg\d
AW
LVIDd
LVIDs
PW
NAC-250mg\kg\d
AW
LVIDd
LVIDs
PW
NAC-500mg\kg\d
AW
LVIDs
LVIDd
PW
(C)
LVEF (%)
ns
NAC-25mg\kg
NAC-250mg\kg
NAC-500mg\kg
(D)
LVFS (%)
NAC-25mg\kg
NAC-250mg\kg
NAC-500mg\kg
(E)
LVIDs (cm)
ns
NAC-25mg\kg
NAC-250mg\kg
NAC-500mg\kg
(F)
LVIDd (cm)
NAC-25mg\kg
NAC-250mg\kg
NAC-500mg\kg
(G)
NAC-25mg/kg/\d
NAC-250mg/kg/d
NAC-500mg/kg/d
MI site
MI site
MI site

Figure S14 (A) Schematic representation of drug administration in mice. (B) Representative M-mode images of the level of the papillary muscles were recorded. (C-F) LVEF and LVFS were relatively increased with increasing NAC concentration. LVIDd and LVIDs in the low-concentration group were higher than those in the high-concentration group. (G) HE staining showing a smaller infarcted area in the myocardium in the high-concentration group than in the low-concentration groups on day 14 after acute MI. Statistical significance was calculated via one-way ANOVA followed by a post hoc Bonferroni test. ns $P > 0.05$, * $P < 0.05$, ** $P < 0.01$; *** $P < 0.001$.

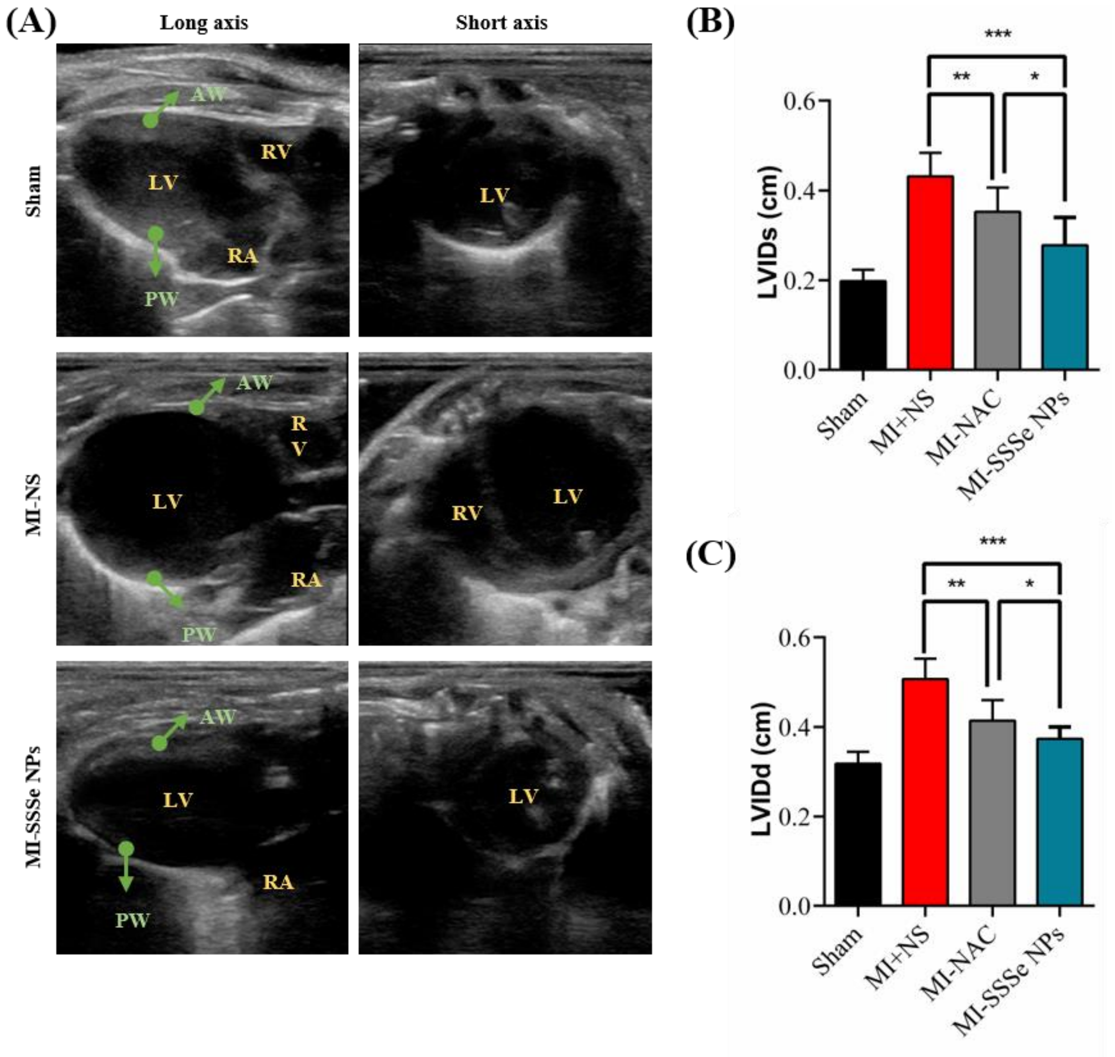

Figure S15 (A) Representative images of echocardiography on day 14 after MI. (B-C) LVIDd and LVIDs in the saline-treated group were higher than those in the NAC-treated group and in the SSSe NP-treated group. Statistical significance was calculated via one-way ANOVA followed by a post hoc Bonferroni test. ns $P > 0.05$, * $P < 0.05$, ** $P < 0.01$; *** $P < 0.001$.

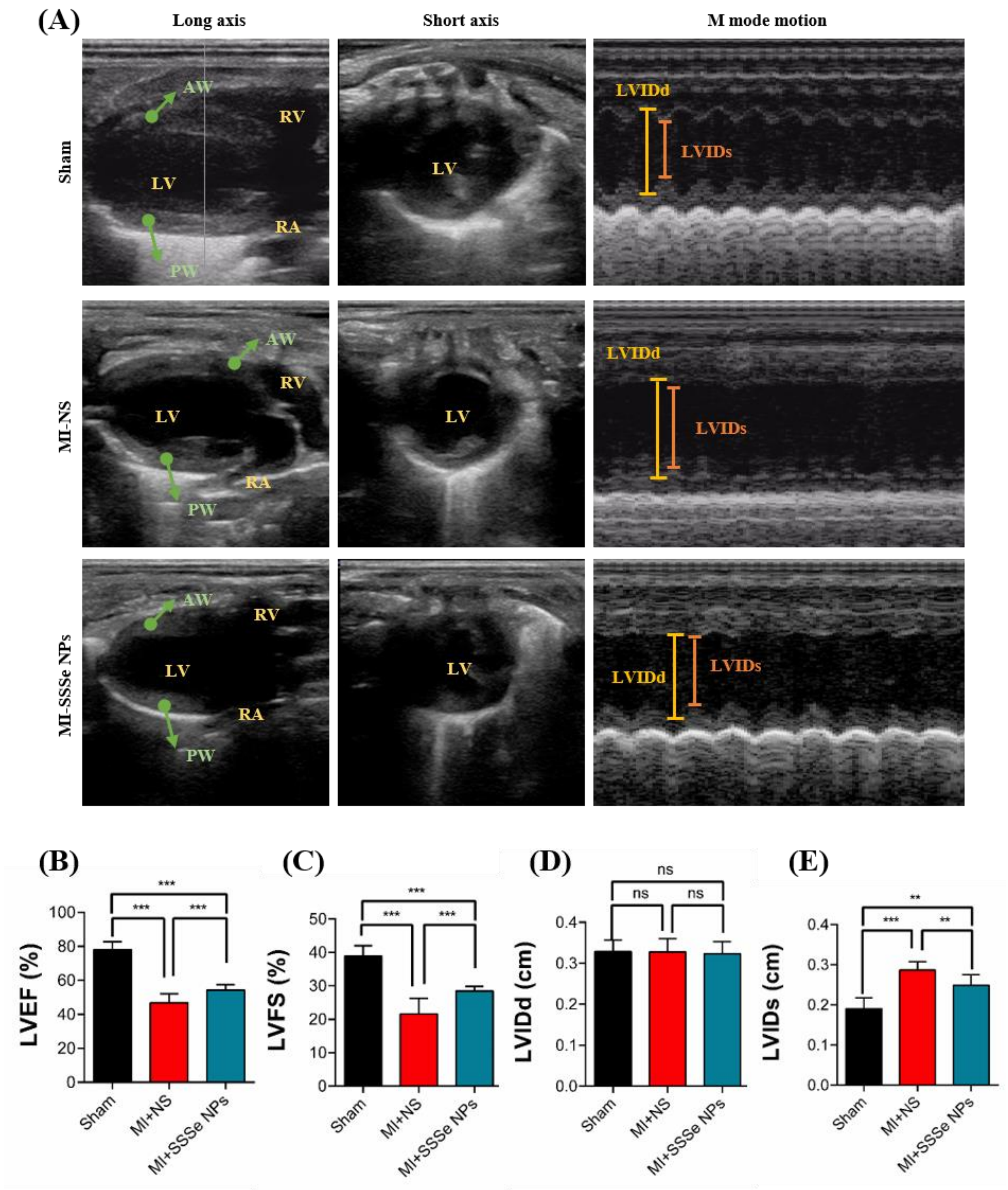

Figure S16 (A) Representative images of echocardiography on day 1 after MI. Representative M-mode images of the level of the papillary muscles were recorded. LVEF: LV ejection fraction; LVFS: LV fractional shortening; LVIDd: LV internal dimension diastolic; LV: left ventricle; RA: right atrium; RV, right ventricle; AW: anterior wall; PW: posterior wall. (B-E) LVEF and LVFS were relatively increased following SSSe NP treatment in comparison with saline treatment. LVIDd and LVIDs in the saline-treated group were higher than those in the SSSe NP-treated group. Statistical significance was calculated via one-way ANOVA followed by a post hoc Bonferroni test. ns $P > 0.05$, * $P < 0.05$, ** $P < 0.01$; *** $P < 0.001$.

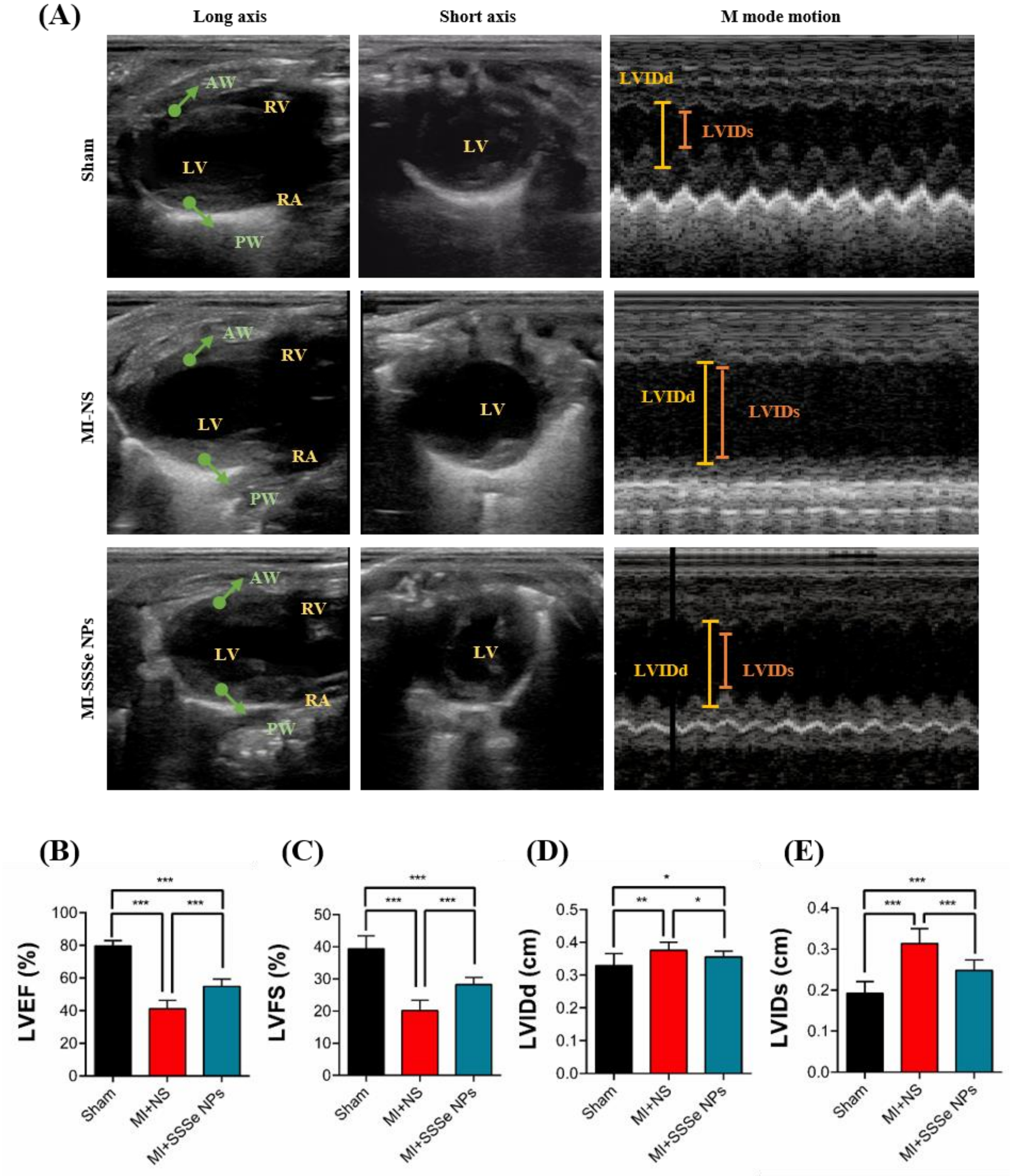

Figure S17 (A) Representative images of echocardiography on day 3 after MI. Representative M-mode images of the level of the papillary muscles were recorded. (B-E) LVEF and LVFS were relatively increased following SSSe NP treatment in comparison with saline treatment. LVIDs and LVIDd in the saline-treated group were higher than those in the SSSe NP-treated group. Statistical significance was calculated via one-way ANOVA followed by a post hoc Bonferroni test. ns $P > 0.05$, * $P < 0.05$, ** $P < 0.01$; *** $P < 0.001$.

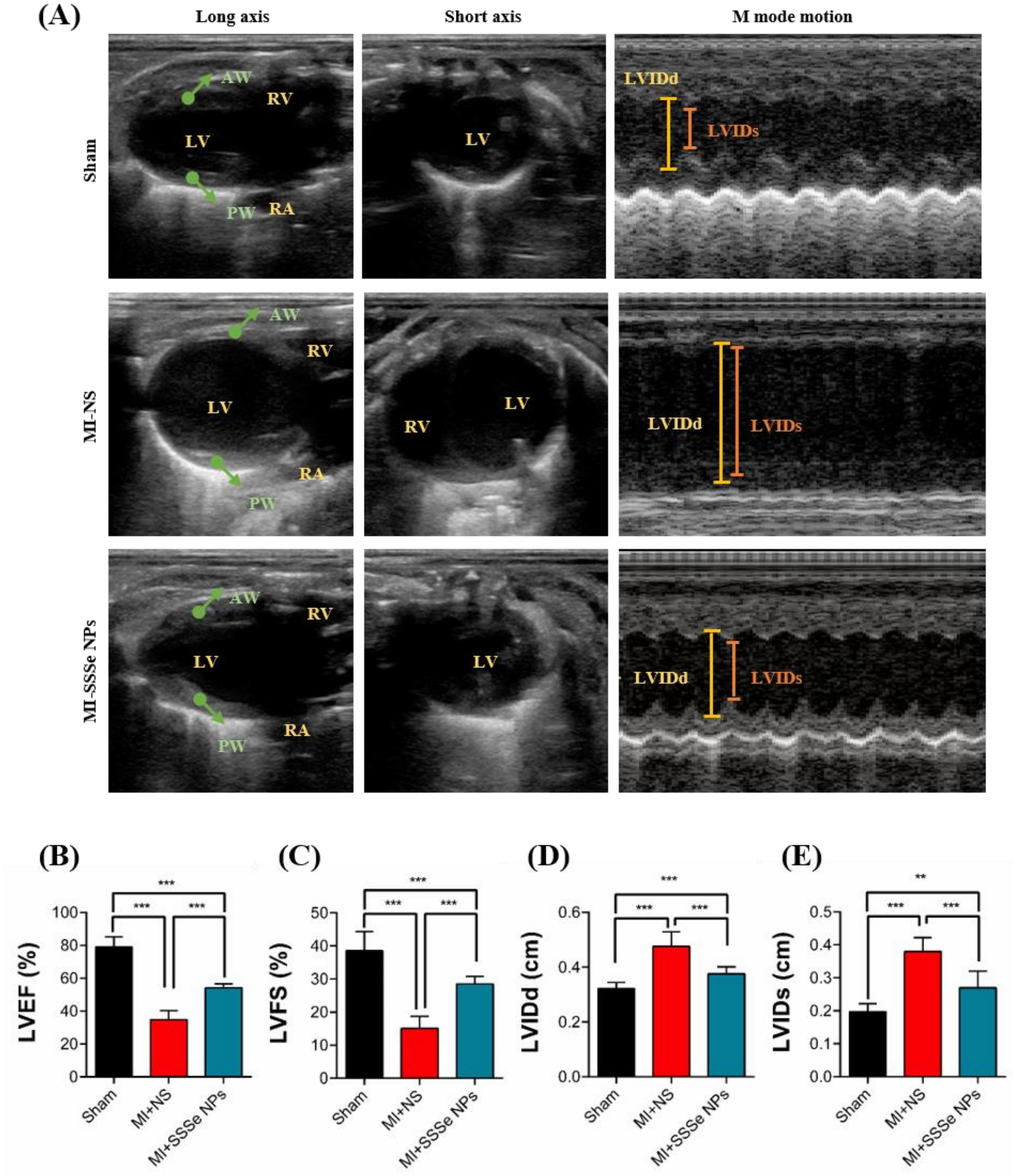

Figure S18 (A) Representative images of echocardiography on day 7 after MI. Representative M-mode images of the level of the papillary muscles were recorded. (B) LVEF and LVFS were relatively increased following SSSe NP therapy in comparison with the saline-treated group. LVIDs and LVIDd in the saline-treated group were higher than those in the SSSe NP-treated group. Statistical significance was calculated via one-way ANOVA followed by a post hoc Bonferroni test. ns $P > 0.05$, * $P < 0.05$, ** $P < 0.01$; *** $P < 0.001$.

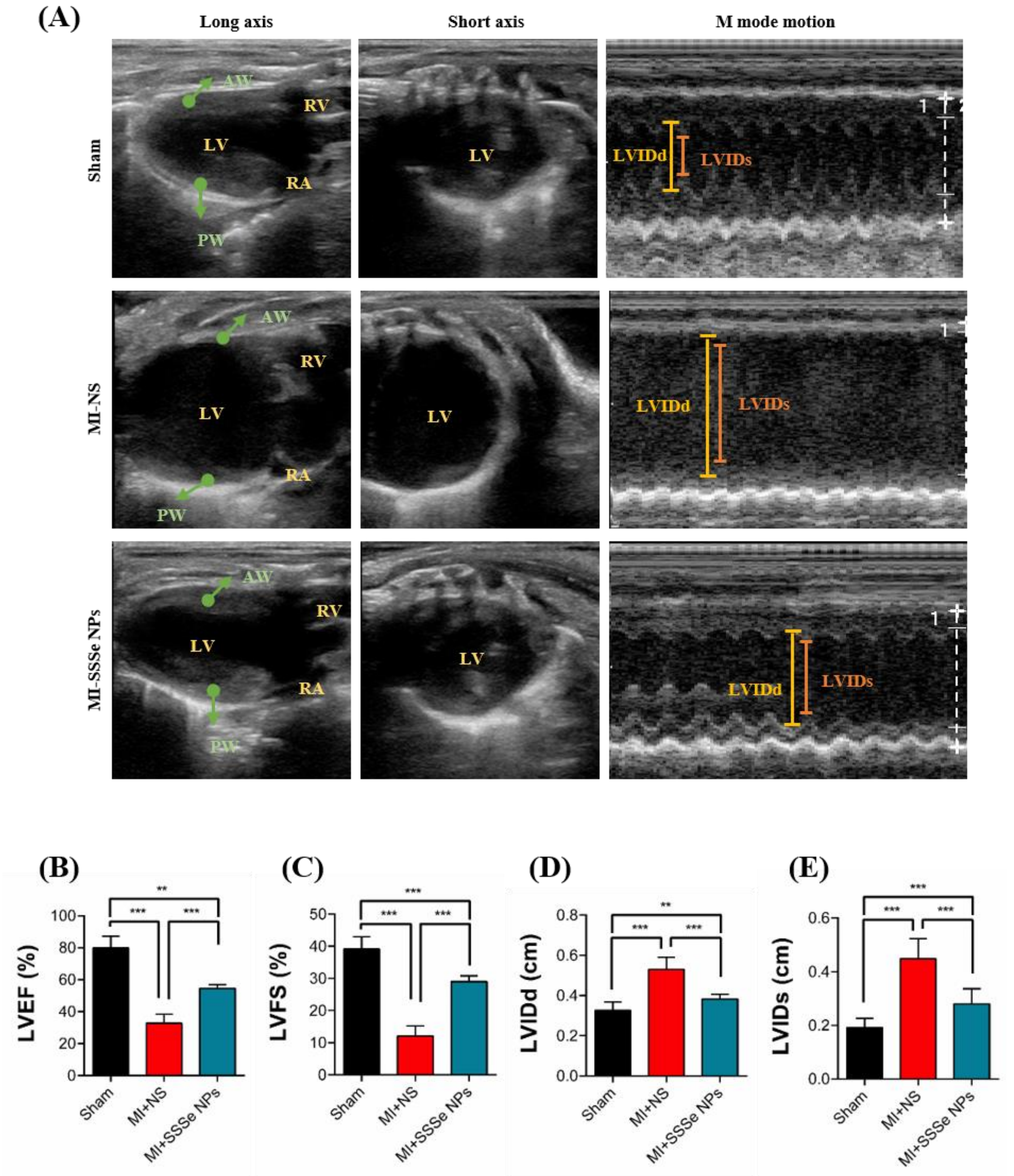

Figure S19 (A) Representative images of echocardiography on day 28 after MI. Representative M-mode images of the level of the papillary muscles were recorded. (B) LVEF and LVFS were relatively increased following SSSe NP treatment in comparison with saline treatment. LVIDd and LVIDs in the saline-treated group were higher than those in the SSSe NP-treated group. Statistical significance was calculated via one-way ANOVA followed by a post hoc Bonferroni test. ns $P > 0.05$, * $P < 0.05$, ** $P < 0.01$; *** $P < 0.001$.

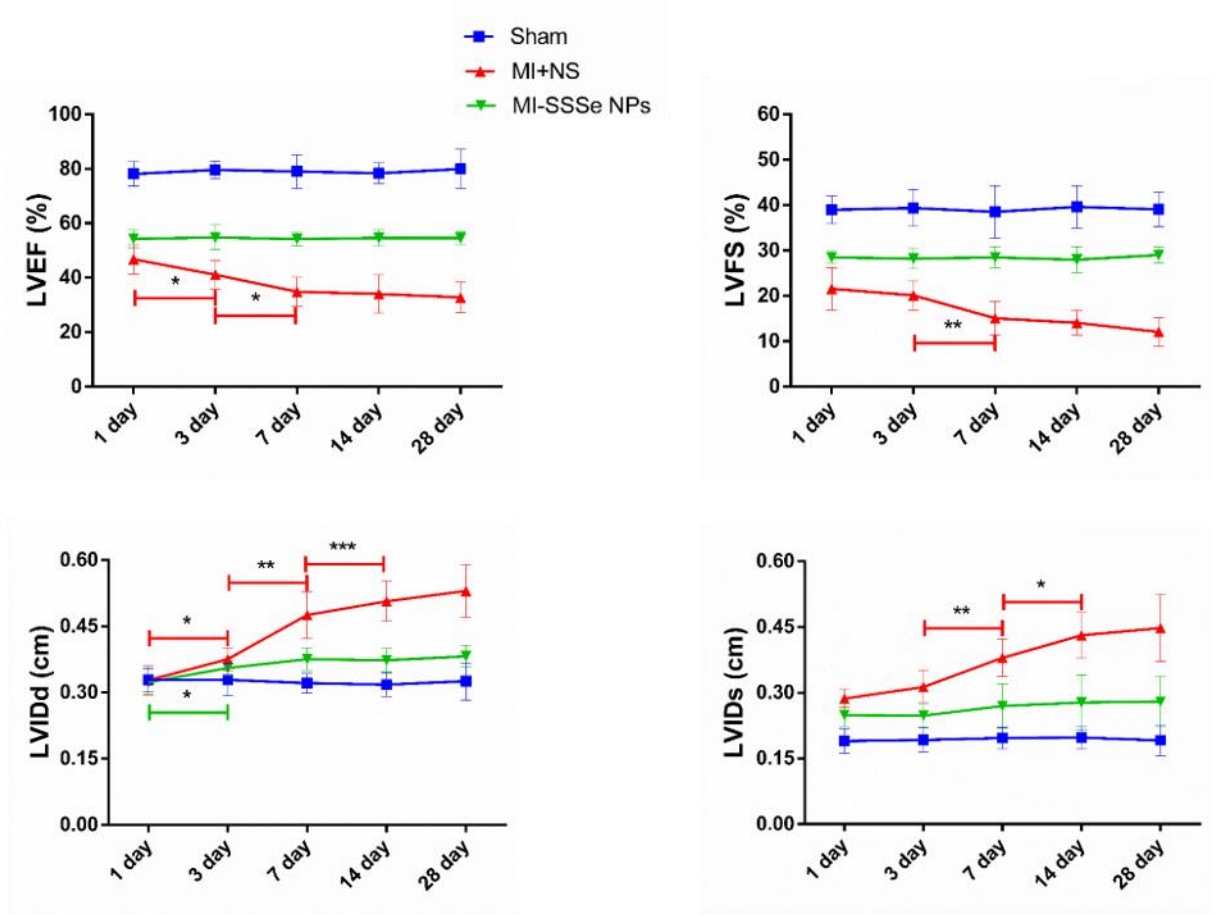

Figure S20 (A-D) The changes in each index of cardiac function (LVEF, LVFS, LVIDd and LVIDs) over time after AMI surgery showing that LVEF and LVFS were greatly decreased and LVIDd and LVIDs were greatly increased on day 7 after MI in saline-treated mice and were maintained lower levels than in the SSSe NP group over time. Statistical significance was calculated via one-way ANOVA followed by a post hoc Bonferroni test. ns $P > 0.05$, * $P < 0.05$, ** $P < 0.01$; *** $P < 0.001$.

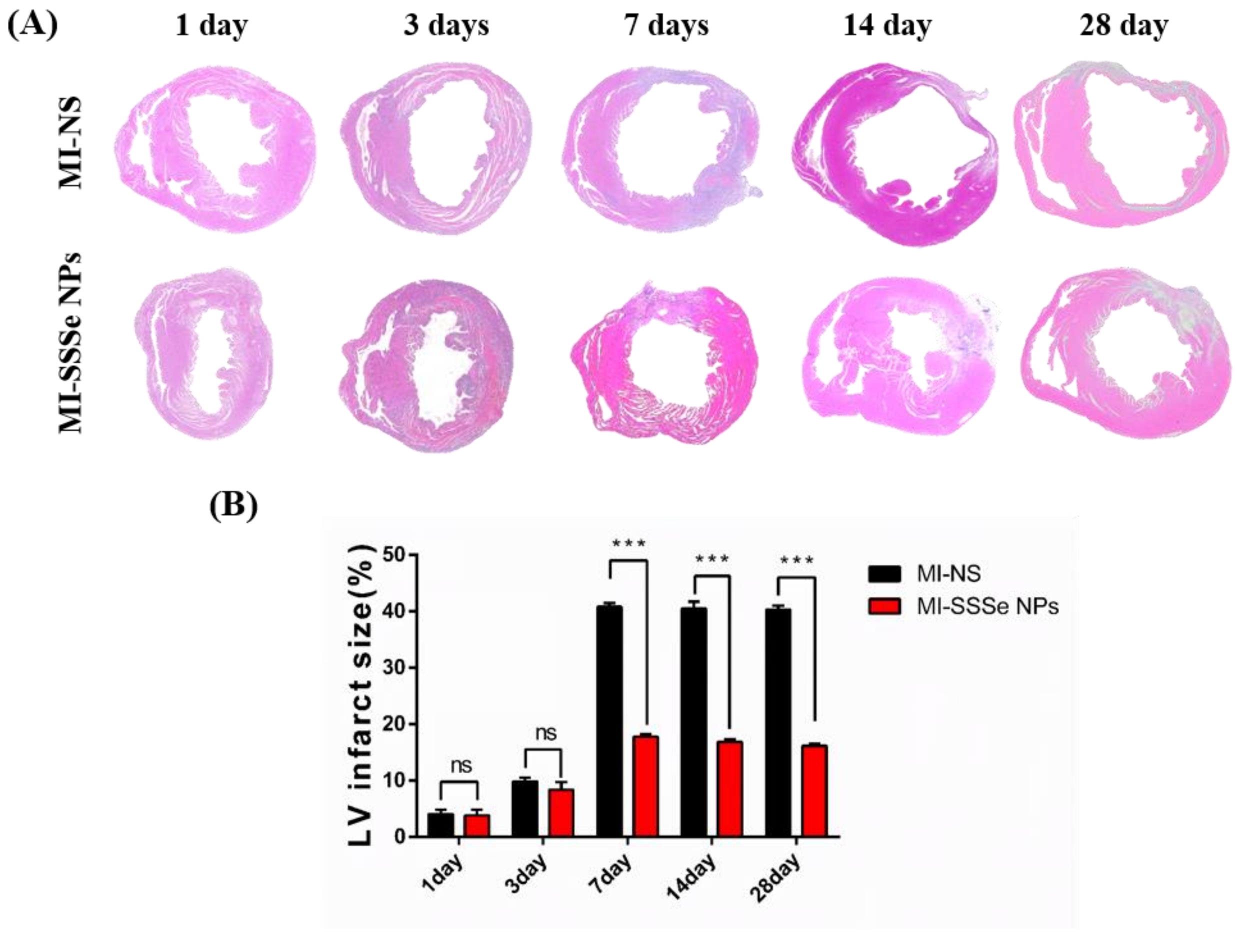

Figure S21 (A) HE staining showing heart restoration after AMI injury in response to SSSe NPs at different time points. (B) Quantification of the percentage of LV infarct size. Statistical significance was calculated by an unpaired two-tailed Student's t test. ns $P > 0.05$, * $P < 0.05$, ** $P < 0.01$; *** $P < 0.001$.

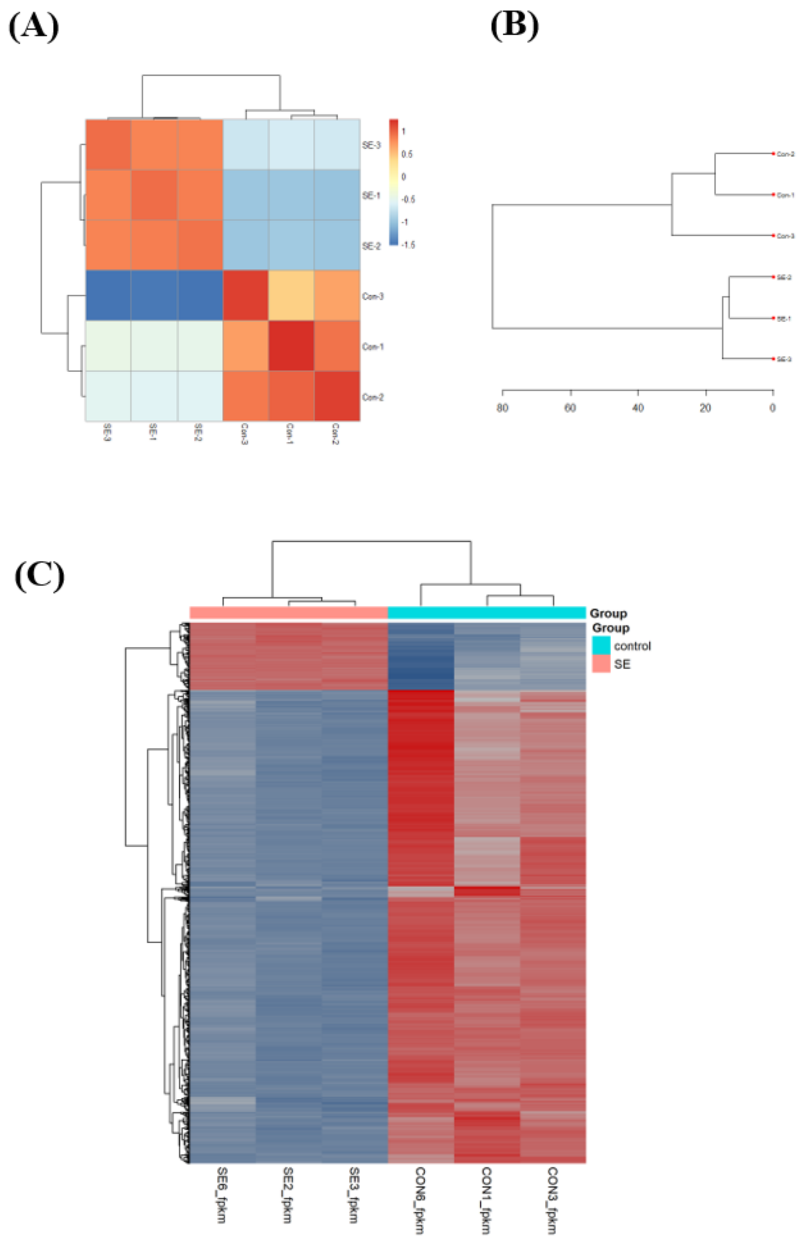

Figure S22 (A) Comparison of Pearson correlations between AMI + NS and AMI + SSSe NP-treated mice. Con: AMI + NS; SE: AMI + SSSe NPs. (B) Cluster analysis between the two groups. (C) Heatmap of DEGs. The legend on the top indicates different groups of genes.

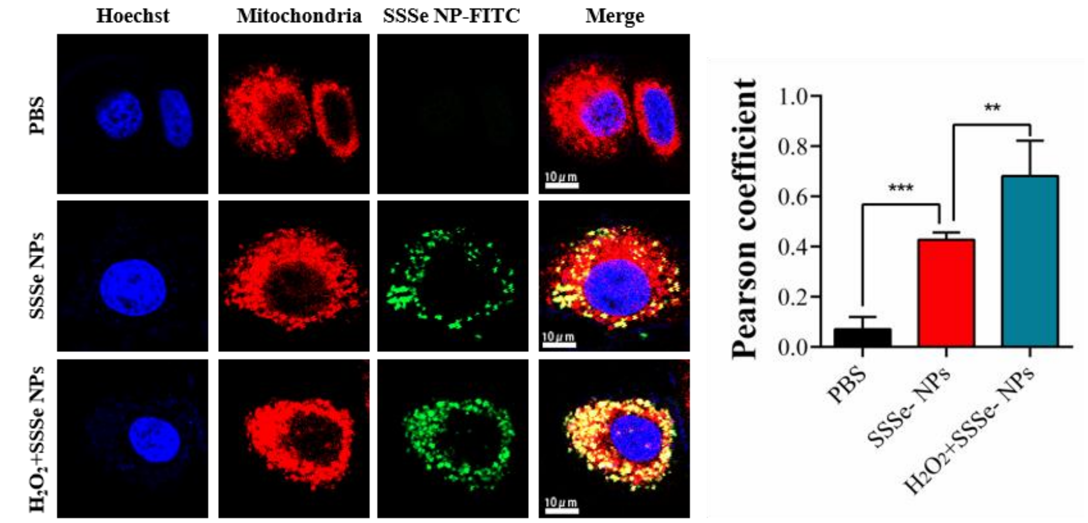

Figure S23 Immunofluorescence colocalization analyses indicated that mitochondria did colocalize with SSSe NP-FITC in cardiomyocytes with (Pearson's Coefficient 0.682) or without (Pearson's Coefficient 0.426) H_2O_2 treatment. a value of 1 indicates total colocalization.

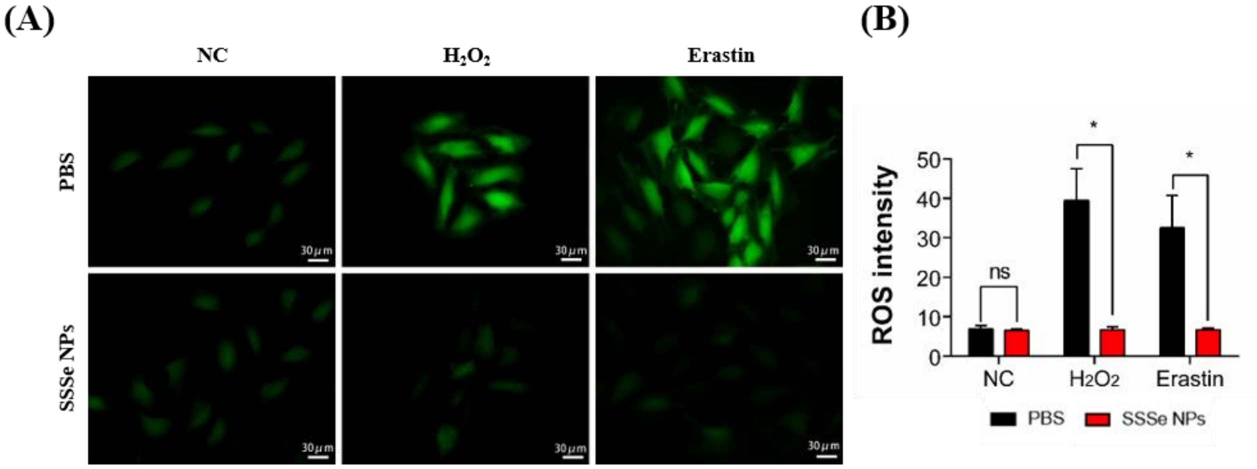

Figure S24 (A) Measurement of intracellular ROS levels using a DCFH-DA probe (green). (B) Quantification of the fluorescence signal intensity of ROS. Statistical significance was calculated by an unpaired two-tailed Student's t test. ns $P > 0.05$, * $P < 0.05$, ** $P > 0.01$; *** $P < 0.001$.

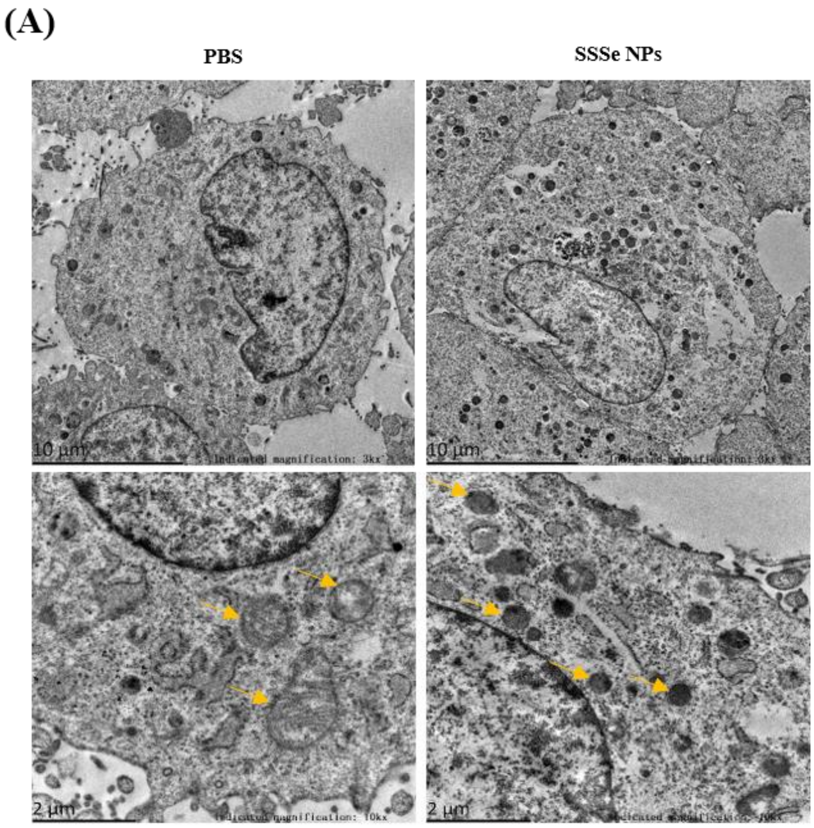

Figure S25 Electron microscopy images showing mitochondrial morphology and cristae in H9C2 cells that were incubated with PBS and SSSe NPs under H_2O_2 stimulation. PBS group: Marked swelling of mitochondria with the presence of giant mitochondria and the formation of different sized vacuoles. SSSe NP group: The cells had a relatively complete mitochondrial structure. (Yellow arrows indicated mitochondria)

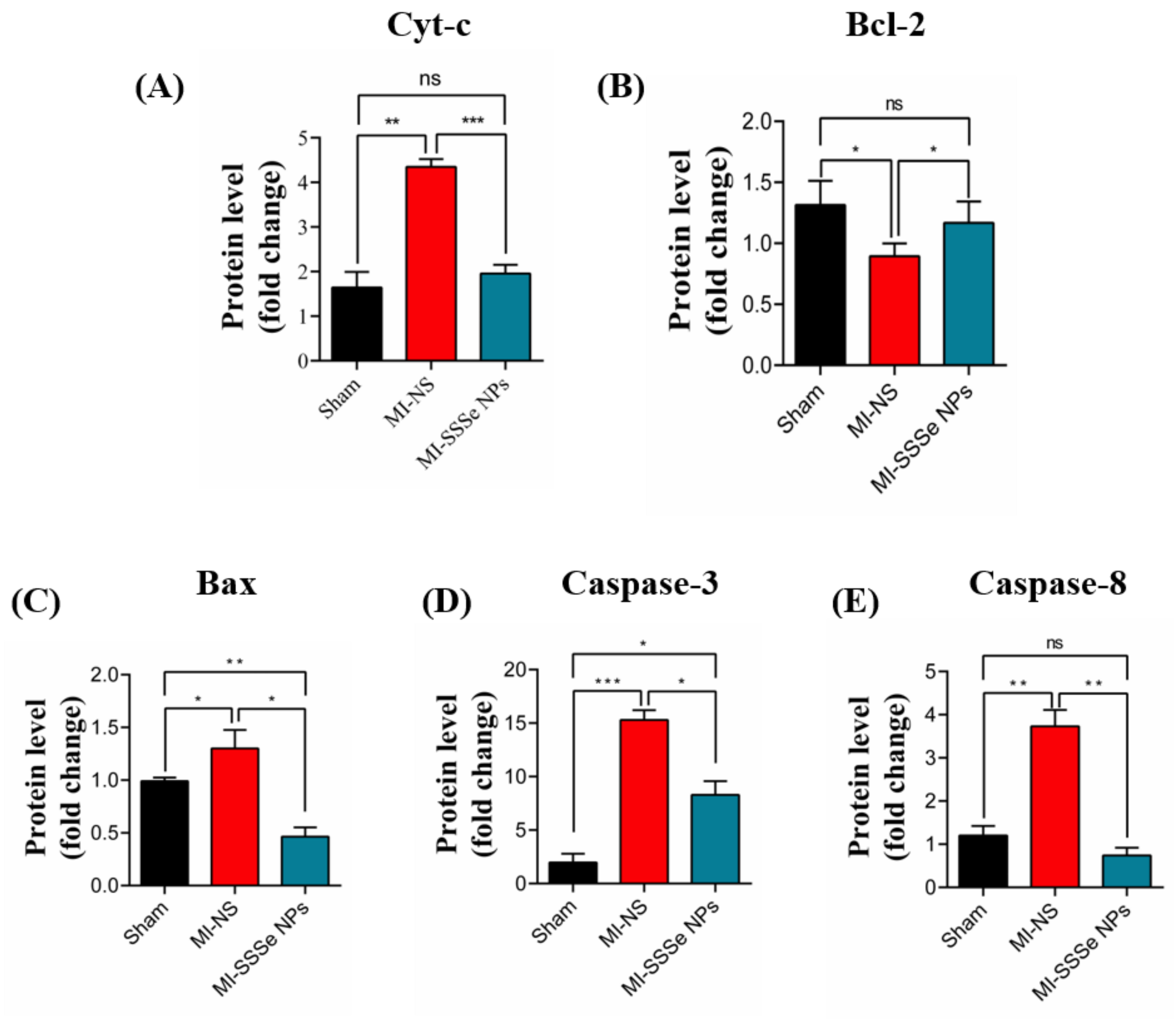

Figure S26 (A-E) Quantification of the relative protein levels (Cyt-c, Bcl-2, Bax, caspase-3 and caspase-8) normalized to β-actin in heart tissues. The results showed that the expression of Cyt-c, Bax, caspase-3 and caspase-8 was significantly higher in the MI-NS group than in the MI-SSSe NP group, while Bcl-2 expression was lower. Statistical significance was calculated via one-way ANOVA followed by a post hoc Bonferroni test. ns $P > 0.05$, * $P < 0.05$, ** $P < 0.01$; *** $P < 0.001$.

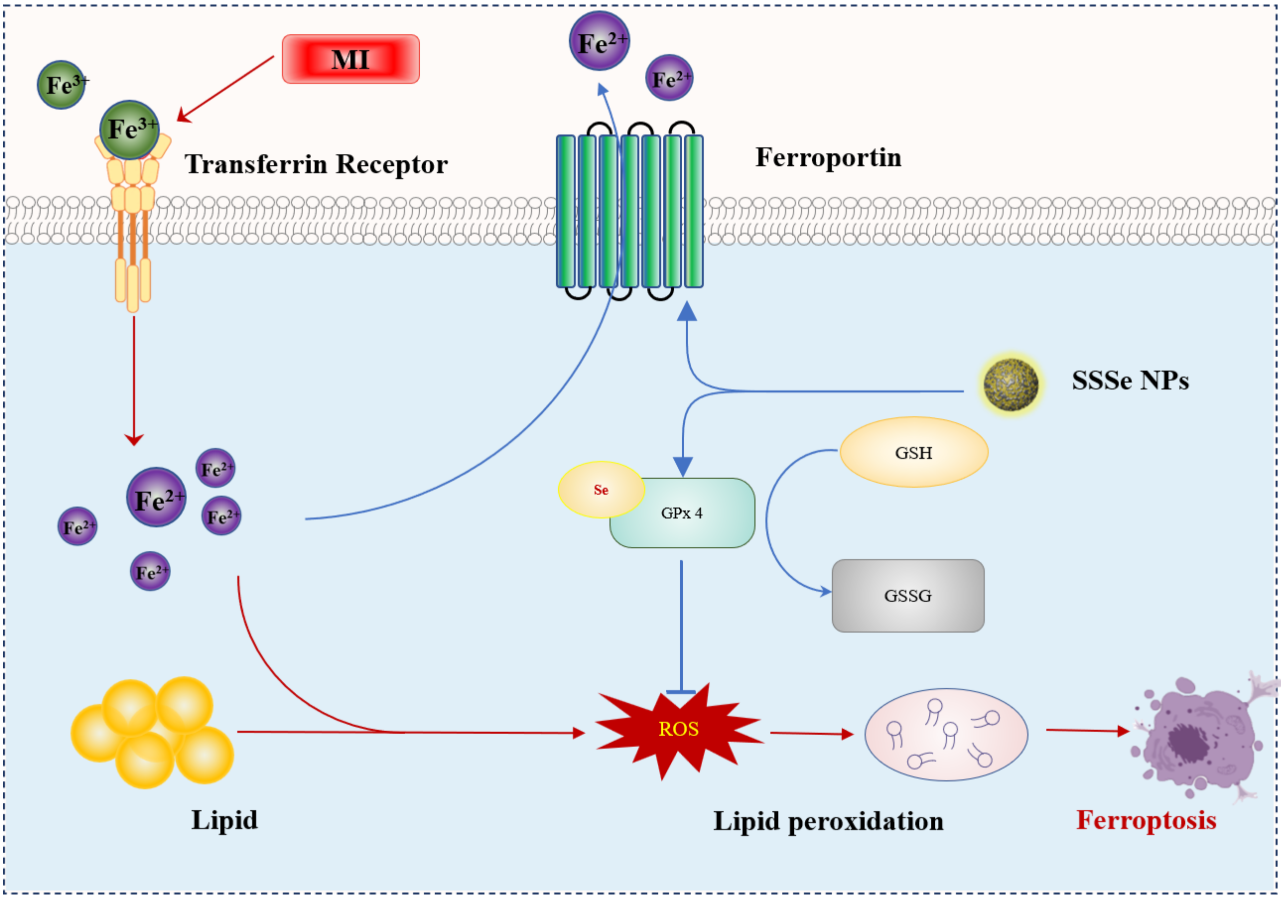

Figure S27 The proposed pathway by which SSSe NPs reduce ferroptosis in myocardial cells through Gpx4 related ROS scavenging and ferroportin related iron transport.

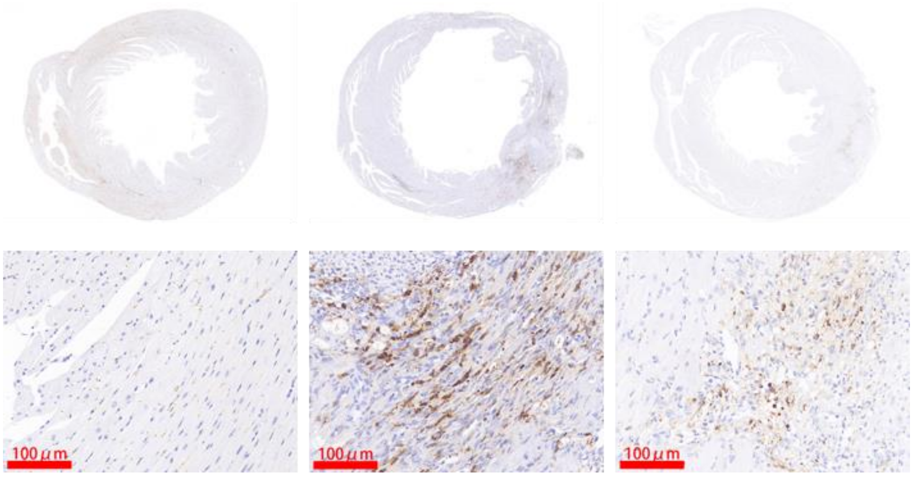

Figure S28 Immunocytochemical analysis of redox-active iron deposits in heart tissues, as measured by a modified Perl's stain.

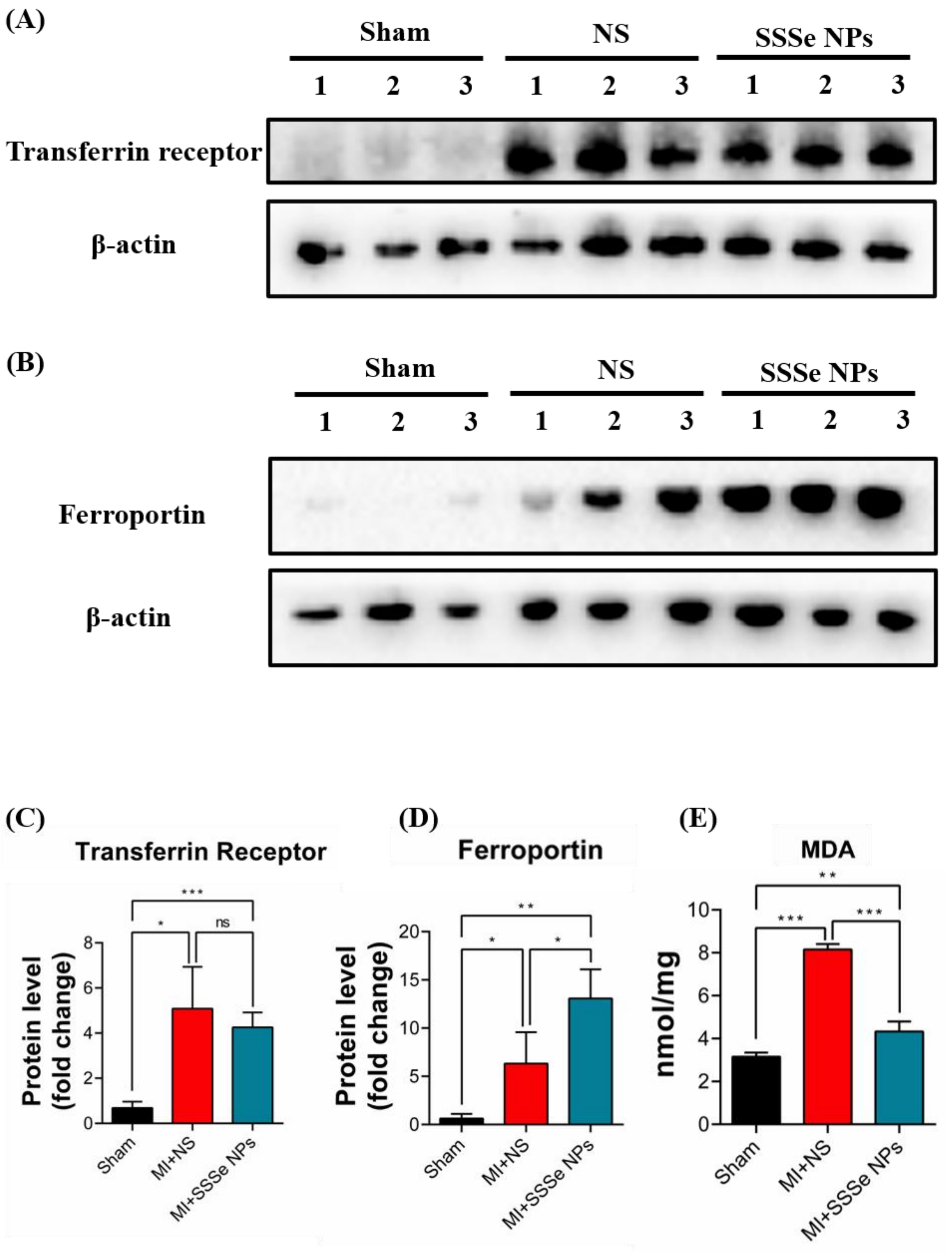

Figure S29 (A-D) Western blot analysis and quantification of the iron transport proteins ferroportin and transferrin receptor. Quantification of the relative protein levels was normalized to β-actin. (E) MDA levels in heart tissues. MDA: malondialdehyde. Statistical significance was calculated via one-way ANOVA followed by a post hoc Bonferroni test. ns $P > 0.05$, * $P < 0.05$, ** $P < 0.01$; *** $P < 0.001$.

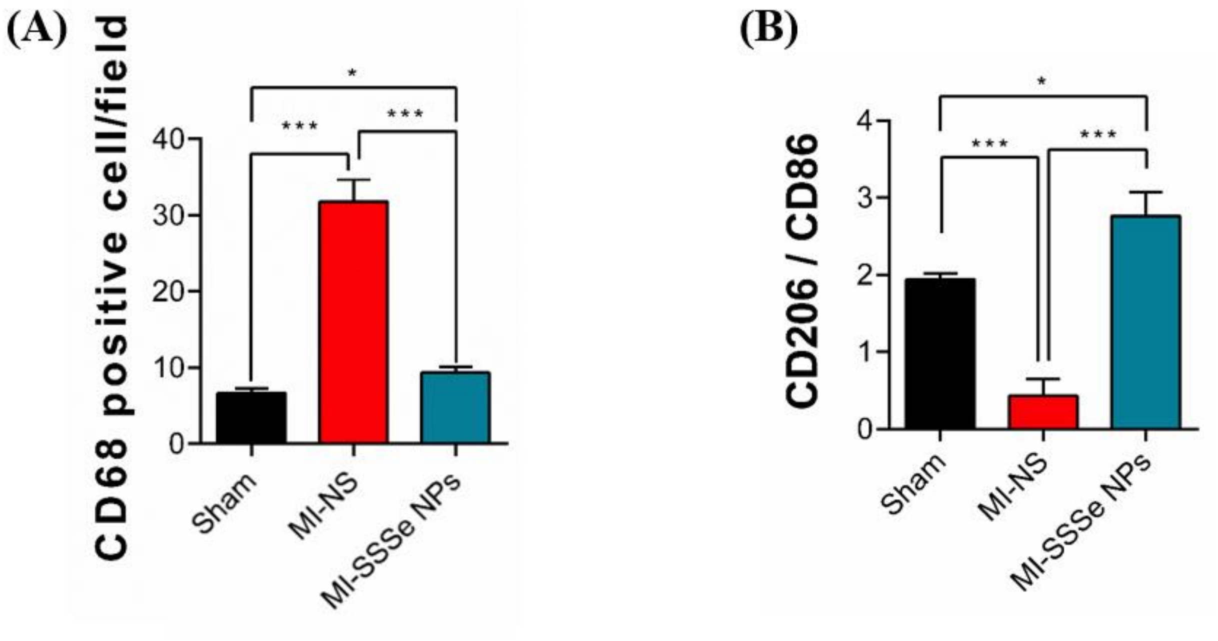

Figure S30 (A) Quantification of the numbers of CD68+ cells in heart tissues. (B) Quantification of the immunohistochemical results. Number of CD206+/CD86+ cells per field in heart tissues.

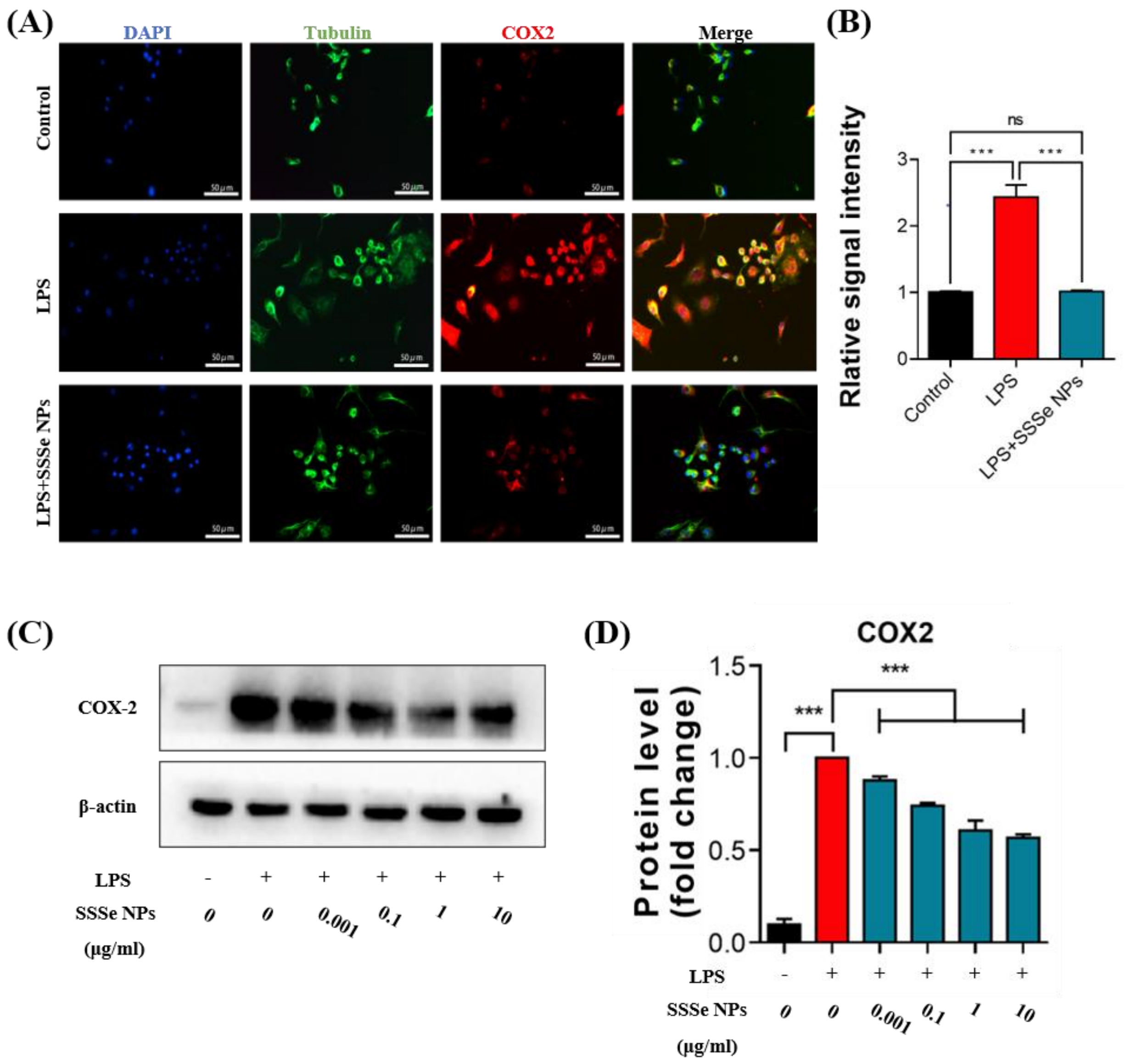

Figure S31 (A) Representative fluorescence images of Tubulin (green), COX-2 (red) and DAPI (blue) in RAW264.7 cells in response to LPS stimulation. Weak COX-2 positive staining was detectable in the SSSe NP group. (B) Quantitation of the relative signal intensity of COX-2$^+$ cells. (C) RAW264.7 cells were pretreated with or without different concentrations of SSSe NPs for 12 h and then treated with LPS for 24 h. Western blot showing the protein expression of COX-2 (M1 macrophage marker). (D) Quantification of the relative protein levels indicated that SSSe NP treatment attenuated the LPS-induced increase in COX-2$^+$ staining in RAW264.7 cells. Statistical significance was calculated via one-way ANOVA followed by a post hoc Bonferroni test. ns $P > 0.05$, * $P < 0.05$, ** $P < 0.01$; *** $P < 0.001$.

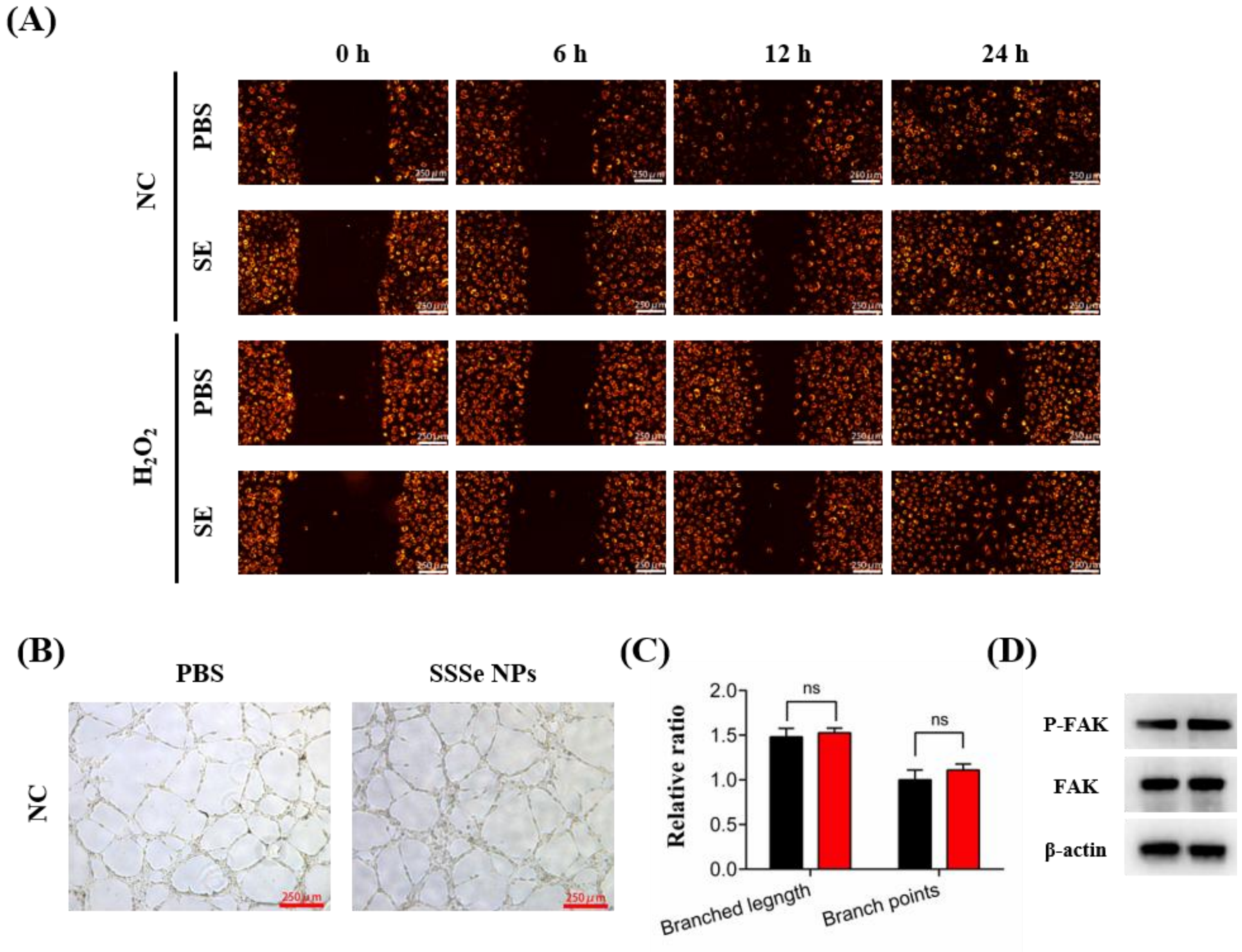

Figure S32 (A) Representative micrographs of the scratch region showing the effect of SSSe NPs on migration under normal conditions and H_2O_2 exposure. (B) Typical images of the tube formation assay. (G) Angiogenesis was measured by an in vitro tube formation assay, and the bar graph shows the percentage of branch lengths and points. All data are presented as the mean ± S.D. (n = 3). (D) Representative Western blot showing p-FAK and FAK expression in HUVECs treated with PBS or SSSe NPs under H_2O_2 injury conditions.

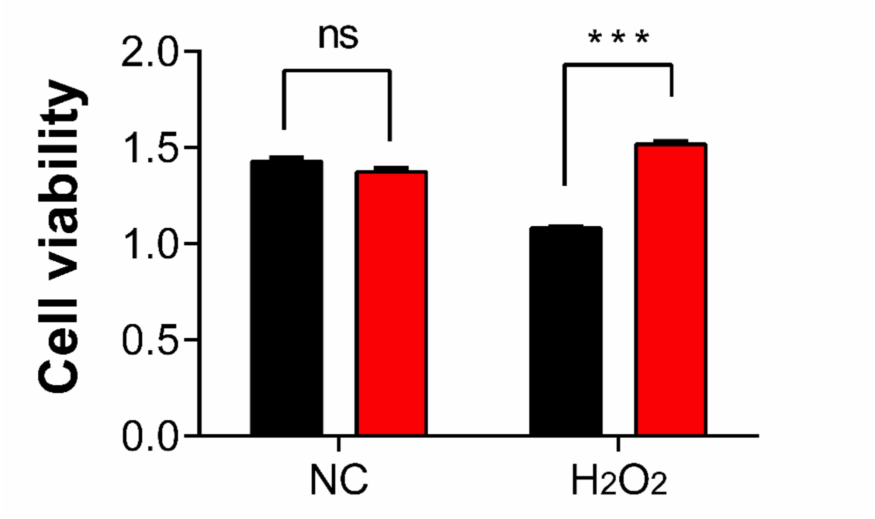

Figure S33 The viability of HUVECs during H_2O_2 stimulation with or without SSSe NPs was assayed by a CCK-8 kit.

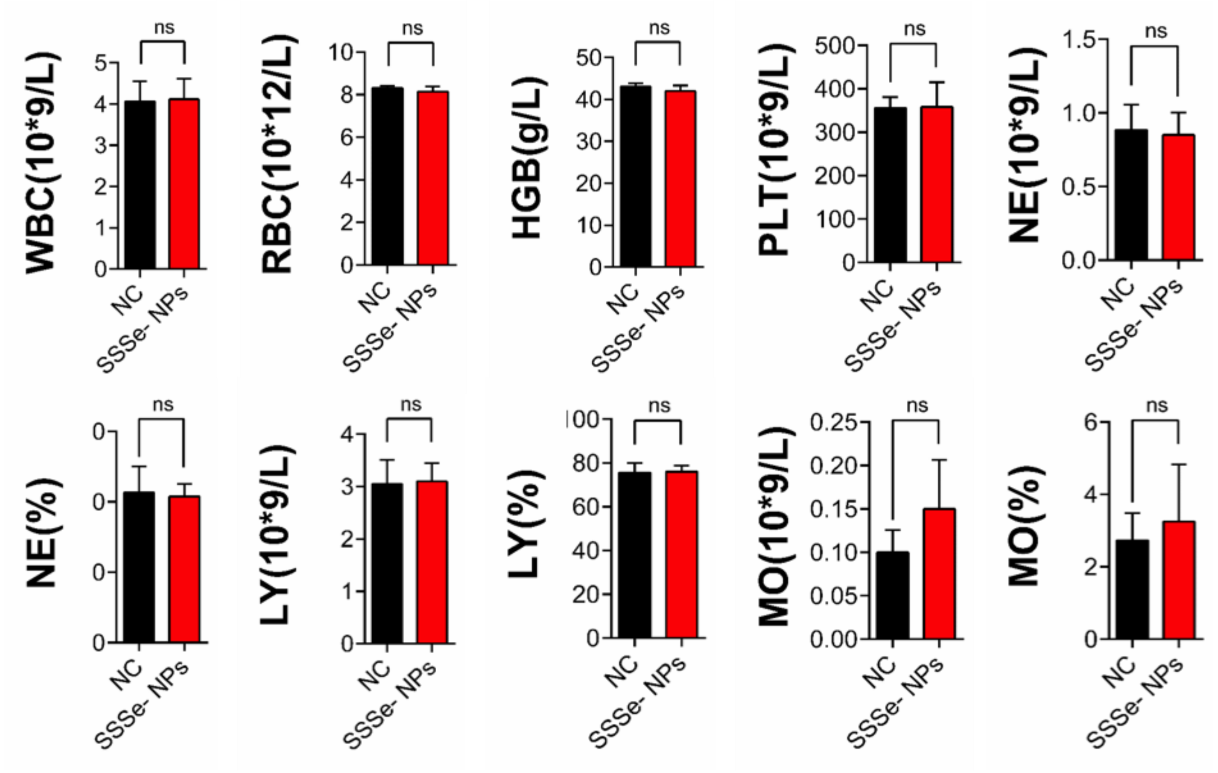

Figure S34 Blood hematology data of mice treated with saline/SSSe NPs after surgery. Blood was collected on day 1 after MI. The data are based on six mice per group. WBC: white blood cell, RBC: red blood cell, HGB: hemoglobin, PLT: platelet, NE: neutrophilic granulocyte, LY: lymphocyte, MO: monocyte. Statistical significance was calculated by an unpaired two-tailed Student's t test. ns $P > 0.05$.

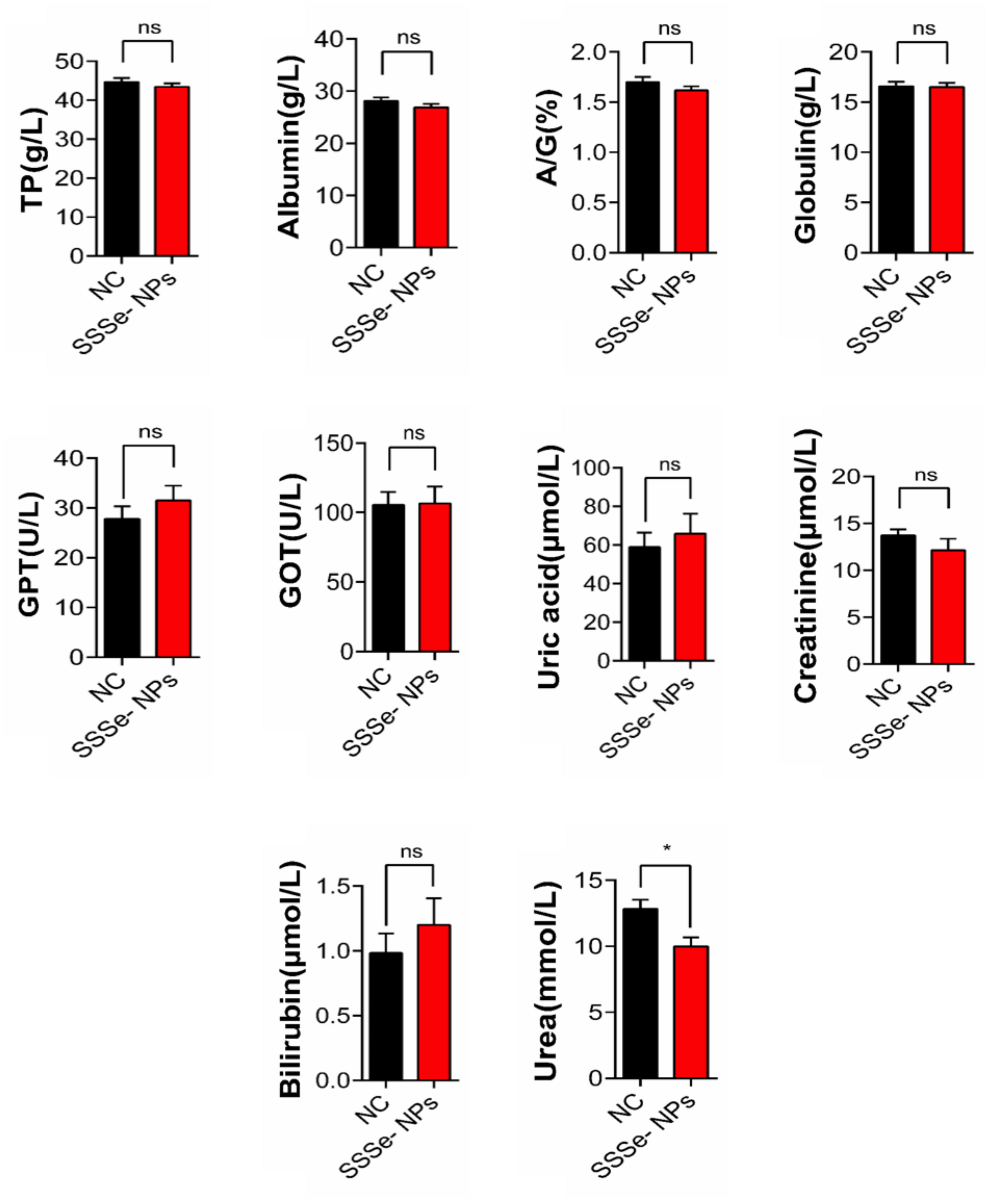

Figure S35 Blood biochemistry data of mice treated with saline/SSSe NPs after surgery. Blood was collected on day 1 after MI. The data are based on six mice per group. TP: total protein, A: albumin, B: globulin, GPT: glutamic pyruvic transaminase, GOT: glutamic oxalacetic transaminase. Statistical significance was calculated by an unpaired two-tailed Student's t test. ns $P > 0.05$, * $P < 0.05$.

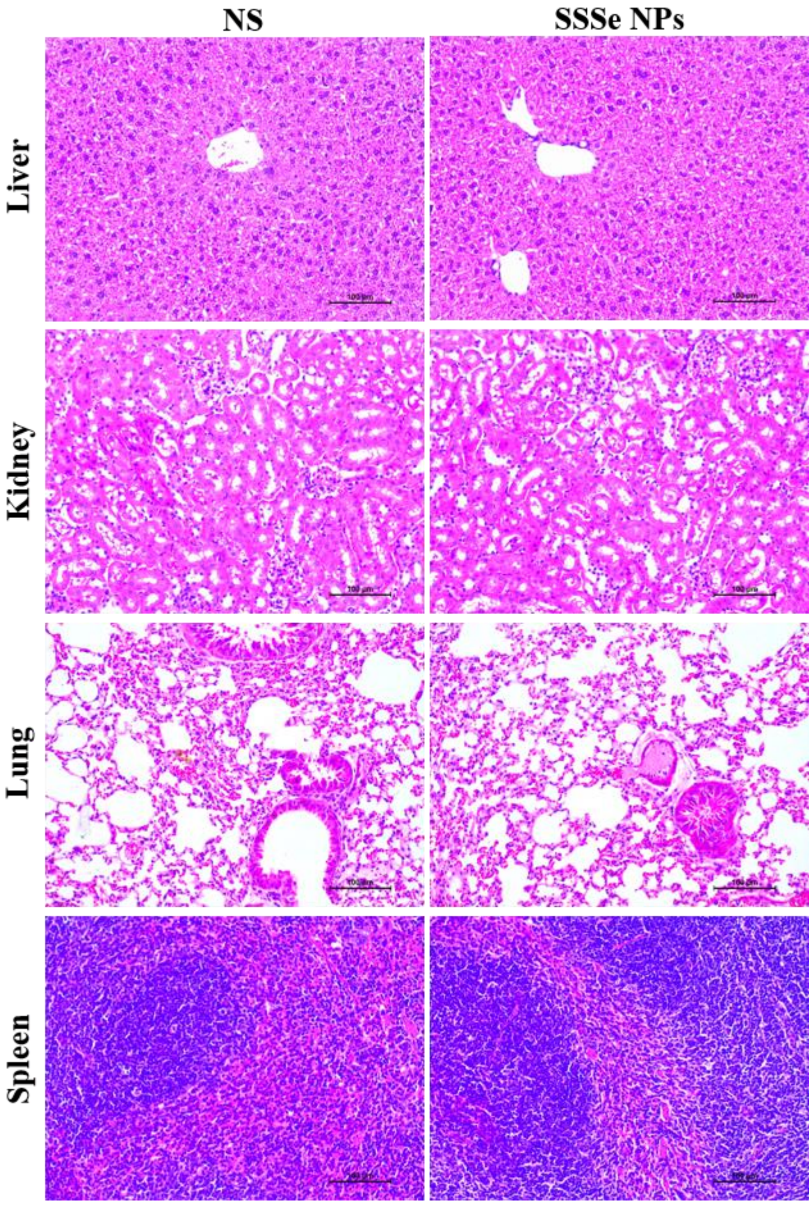

Figure S36 Representative images of HE-stained lung, liver, kidney, and spleen sections from mice. The organs were collected on day 3 after the mice were injected with saline/SSSe NPs.
